# CX3CR1 Modulates Migration of Resident Microglia Towards Cortical Laser‐Induced Lesions

**DOI:** 10.1002/glia.70207

**Published:** 2026-08-15

**Authors:** Jens Wagner, Henrike Antony, Cornelia Hoyer, Kristiina Lundgrén, Rabah Soliymani, Sophie Crux, Lena Justus, Kevin Keppler, Julia Steffen, Christian Kurts, Daniel R. Engel, Jochen Herms, Maciej Łałowski, Martin Fuhrmann

**Affiliations:** ^1^ German Center for Neurodegenerative Diseases (DZNE) Bonn Germany; ^2^ Helsinki Institute of Life Science (HiLIFE), Meilahti Proteomics Unit (MPU) University of Helsinki Helsinki Finland; ^3^ Center of Neuropathology and Prion Research Ludwig Maximilians University Munich Germany; ^4^ Institute of Molecular Medicine and Experimental Immunology, University of Bonn Bonn Germany; ^5^ Institute of Experimental Immunology and Imaging, University Duisburg‐Essen Duisburg Germany; ^6^ German Center for Neurodegenerative Diseases (DZNE) Munich Germany; ^7^ Department of Gene Expression Institute of Molecular Biology and Biotechnology, Faculty of Biology, Adam Mickiewicz University Poznań Poland

**Keywords:** CX3CR1, cytoskeleton, microglia, migration

## Abstract

Microglia are innate immune cells of the central nervous system (CNS). They extend their processes and migrate toward injuries in vivo. However, how the fractalkine receptor (CX3CR1) influences microglial migration remains unknown. Label‐free proteomic profiling predicted changes in Ras homology family (RHO)‐signaling activity that hint at dysregulated cytoskeleton signaling in *Cx3cr1‐*deficient murine cortex tissue. To further investigate microglial migration, we carried out two‐photon in vivo imaging at 4‐h intervals for 72 h after a laser lesion in the cortex. *Cx3cr1*‐deficient microglia showed enhanced migration toward the lesion. Additionally, the length and velocity of microglial fine processes extending toward the lesion were increased in *Cx3cr1‐*deficient microglia. Migration remained unchanged in *Ccr2*‐deficient mice, indicating that monocyte‐derived macrophages/microglia did not contribute to microglia accumulation around the lesion. These results demonstrate that CX3CR1 modulates microglia migration toward laser‐induced CNS injury. Manipulating microglia migration via the CX3CR1 signaling axis is therefore a potential target for the treatment of CNS injury.

## Graphical abstract

CX3CR1 deficiency promotes microglial migration toward CNS injury. Proteomic profiling reveals altered RHO‐mediated cytoskeletal signaling, while in vivo two‐photon imaging demonstrates increased process extension, velocity, and accumulation of Cx3cr1‐deficient microglia at lesion sites.

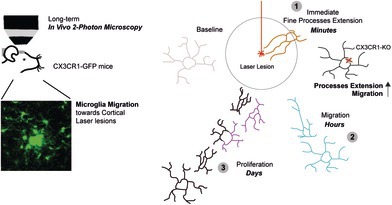

## 1 Introduction

Microglia are mononuclear phagocytes that reside in the parenchyma of the central nervous system (Ransohoff and Cardona 2010). During embryogenesis, yolk sac‐derived microglia colonize the brain, while after this critical period, microglia persist in the CNS without substantial input from bone‐marrow‐derived myeloid cell populations under healthy conditions anymore (Ginhoux et al. 2010; Kierdorf et al. 2013; Ajami et al. 2007). It is hypothesized that resident microglia proliferate to maintain a stable population. However, under specific neurodegenerative conditions, myeloid precursors may enter the brain via an impaired blood–brain barrier (BBB) (Prinz et al. 2011). Microglia are highly motile cells with long processes that constantly scan the surrounding brain parenchyma (Nimmerjahn et al. 2005; Davalos et al. 2005).

Microglia respond to injuries in the CNS. In the healthy brain, only 5% of microglia are mobile; however, this number increases in a sex‐dependent manner in response to brain hemorrhages (Boghozian et al. 2023). Upon brain injury, microglia extend their processes toward a lesion. Within 30 min, processes of microglial cells in proximity reach the damaged site, which is followed by chemotaxis of microglia toward the injury (Davalos et al. 2005; Askew et al. 2017). This migration process is driven by adenosine triphosphate (ATP) via the purinergic G protein‐coupled P2Y receptor (Davalos et al. 2005). The metabotropic P2Y_12_ receptor is suggested as the major site where ATP induces microglial chemotaxis (Haynes et al. 2006). Indeed, ATP, which is released at the site of brain damage, induces microglial migration (Zahiri et al. 2021; Gyoneva and Traynelis 2013). Further, microglial accumulation in and around a diphtheria toxin A‐chain‐expression‐induced lesion was shown (Rice et al. 2015). Moreover, neuronal lesions in the brain do not induce migration of peripheral cells into the brain, concluding that the BBB remains intact. Therefore, cells accumulating in and around a laser lesion are presumably microglia and not peripheral myeloid cells, such as monocytes or macrophages (Rice et al. 2015). Not only at sites of brain injury is accumulation of microglia detected, but also around lesions of Multiple Sclerosis (MS) pathology (Baaklini et al. 2023; Dong et al. 2021). In MS, microglia are also associated with a higher lesion load (van den Bosch et al. 2024).

One key receptor involved in microglial migration and chemotaxis is the fractalkine receptor (CX3CR1) and its ligand, fractalkine (CX3CL1). Specifically, Lipopolysaccharide (LPS)‐stimulated intracranially injected *Cx3cr1* ^*−/−*^ microglia do not migrate along white matter tracts (Cardona et al. 2006). In addition, increased microglial migration toward dying neurons in an Alzheimer's Disease (AD) mouse model is rescued by *Cx3cr1* knockout, further supporting a role for CX3CR1 in migration (Fuhrmann et al. 2010). Thus, microglial *Cx3cr1*‐deficiency can be either detrimental or beneficial depending on the lesion context (Cardona et al. 2006; Fuhrmann et al. 2010; Bhaskar et al. 2010). Directed migration of Cx3cr1‐eGFP labeled microglia toward a microhemorrhage over a time interval of 48 h in vivo has been previously shown (Ahn et al. 2018), however, the role of *Cx3cr1* in regulating microglial movements remains elusive.

To better understand the role of *Cx3cr1* in microglial dynamics in response to a cortical laser lesion under sterile inflammatory conditions, we investigated *Cx3cr1*‐mediated microglial migration and proliferation upon induction of a laser lesion using two‐photon in vivo imaging. We performed unprecedented temporal resolution 4 h time‐interval imaging for a period of up to 72 h to monitor microglial dynamics. We also analyzed whether peripheral monocyte‐derived macrophages and proliferating microglia contributed to the local accumulation of cells around the lesion. Our results support the conclusion that primarily resident microglia accumulate at a lesion, and CX3CR1 modulates microglial migration and process motility.

## 2 Results

### 2.1 Proteomic Profiling Reveals Differential Expression of Cytoskeleton Signaling Proteins

**FIGURE 1:**
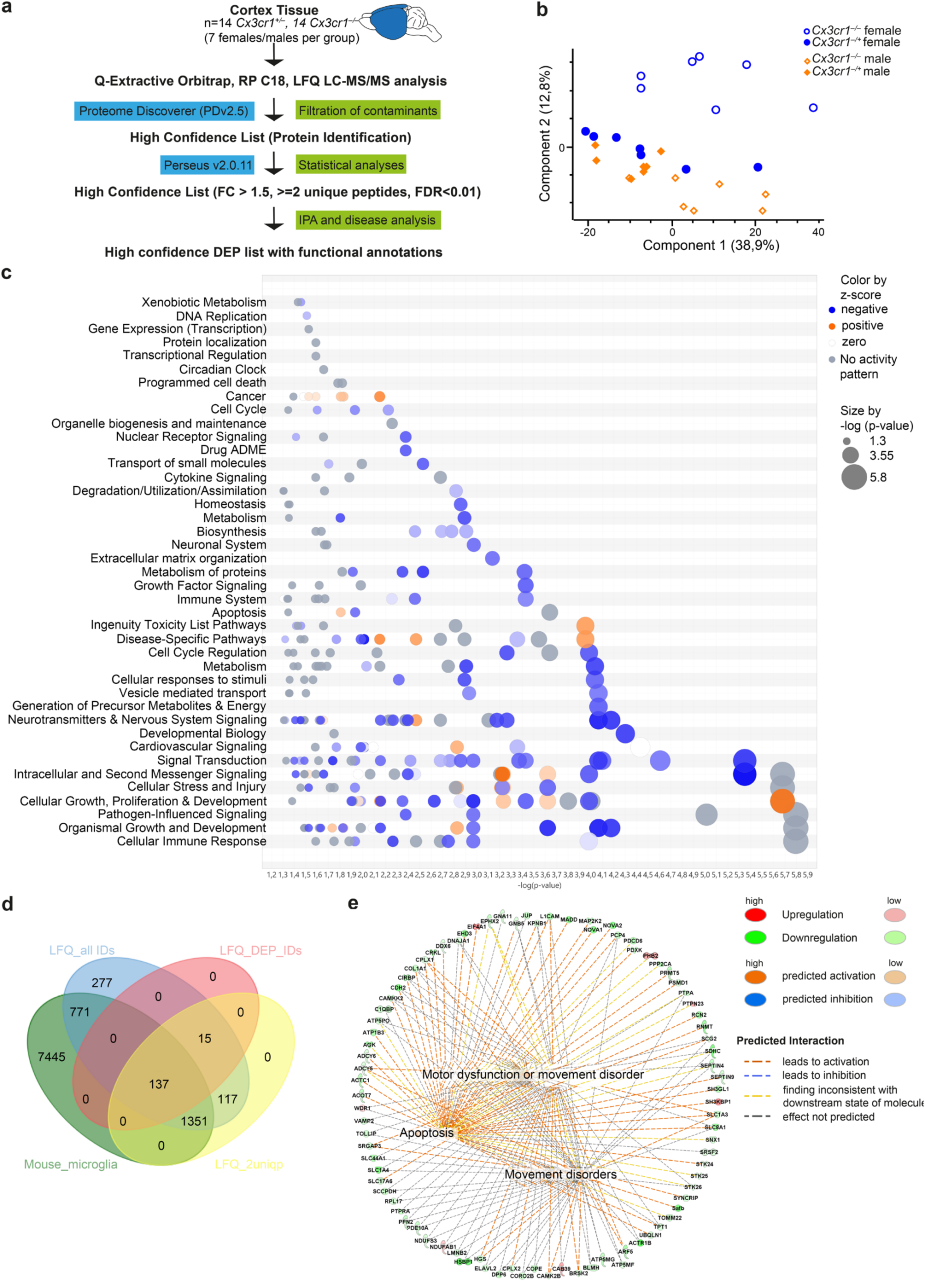
Global comparison of *Cx3cr1* ^+/−^ and *Cx3cr1* ^−/−^ proteomes in the cortex. (a) Workflow of the LFQ proteomics analysis used to determine alterations in protein abundance between *Cx3cr1* ^−/−^ and *Cx3cr1* ^+/−^ mice. (b) Unsupervised PCA to evaluate the overall separation between *Cx3cr1* ^−/−^ (orange) and *Cx3cr1* ^+/−^ (blue) male and female mice. Analysis was performed using a high‐confidence set of proteins (identified with ≥ 2 unique peptides at FDR < 0.01). (c) Canonical pathways related to differentially abundant proteins (DEPs). (d) Venn diagram comparing all identified cortical proteins (LFQ_all IDs), high‐confidence proteins (≥ 2 unique peptides, FDR < 0.01), DEPs (LFQ_DEP_IDs), and a microglial proteome dataset (Mouse_micro; (Lloyd et al. 2024)). (e) Disease‐and‐function network with predicted relationships. Please find an enlarged version of Figure 1e for improved readability in the Supporting Information (Figure S8).

To identify protein expression changes associated with CX3CR1 deficiency, we performed label‐free quantitative proteomic profiling of the cortex, comparing *Cx3cr1* ^*−/−*^ and *Cx3cr1* ^*+/−*^ mice. The analysis aimed to elucidate alterations in protein abundance and identify disrupted biological pathways or local networks. Whole‐cortex tissue samples were processed, allowing the identification of a total of 2668 proteins at FDR < 0.05 (Figure 1a, Table S1). To assess group similarity, we first performed a Principal Component Analysis (PCA) on a high‐confidence protein subset (identified by at least two unique peptides; FDR < 0.01). Initial PCA indicated a clear separation between the four experimental groups based on genotype and sex (Figure 1b). To isolate genotype‐driven effects, we implemented a two‐way ANOVA with genotype and sex as independent factors, effectively controlling for sex‐specific variance and genotype‐sex interactions. Significance thresholds were set at an ANOVA *p*‐value < 0.05, and a minimum absolute log2 fold change of 0.58 (1.5‐fold change). Following this filter, an updated PCA of the 152 genotype‐significant proteins (differentially abundant proteins, DEP) revealed that genotype was the primary driver of variance (PC1 = 55.7%), while PC2 (14.7%) captured sex‐specific divergence (Figure S1a and Table S2). Furthermore, hierarchical clustering (Spearman correlation, average linkage) of this protein signature demonstrated high consistency, with 92.6% (25/27) of biological replicates clustering accurately by genotype (Figure S1d). The proteomic findings were contextualized by comparing 152 DEPs with the Lloyd et al. (Lloyd et al. 2024) microglial proteome database, confirming 137 DEPs (90.1%) and 1488 of 1620 high‐confidence proteins (91.9%, identified at the level of 2 unique peptides, and FDR < 0.01; 91.9%) as established microglial members (Figure 1c and Table S4). Comparison with transcriptomic datasets (Gyoneva et al. 2019; Zhang et al. 2014; Butovsky et al. 2014; Hickman et al. 2013) revealed minimal overlap, with only two proteins (Kctd12, Slc1a3) shared with Butovsky et al. (Figure S1b).

Interestingly, the proteomes of animals from the two genotypes differed significantly between the experimental groups based on analysis using an ANOVA test as described in the methods section, hinting at functional differences. Based on the identified DEPs, we performed Ingenuity Pathway Analysis (IPA) to predict alterations in cellular signaling processes (Table S3). Out of all 631 canonical pathways, 81 of them were allotted a z‐score, with 2 pathways predicted to be upregulated, and the majority to be downregulated (Figure 1d and Figure S1c), including various signaling cascades and cytoskeletal alterations. We observed significant dysregulation of RHO‐signaling pathways (i.e., Signaling by Rho Family GTPases, *z*‐score = −3162) involving cytoskeletal changes between the groups, indicating that many migration‐regulating signaling processes were downregulated in *Cx3cr1* ^*−/−*^ compared to *Cx3cr1* ^*+/−*^ mice. RHO‐specific guanine nucleotide dissociation inhibitors (RHOGDIs) maintain the inactive fraction of RHO‐GTPases in the cytosol, while only a few are active in the cytoplasm (Garcia‐Mata et al. 2011). RHO‐GTPases were found to modulate many cellular processes, including migration, as well as cytoskeletal regulation (Hodge and Ridley 2016). CX3CR1 is expressed in microglia and cortical perivascular/meningeal macrophages in the CNS; however, expression of *Cx3cr1* mRNA is highest in microglial cells (Goldmann et al. 2016). Since CX3CR1 is most prominently expressed on microglia in the cortex, it implies a specific modulation of microglial cytoskeletal signaling, potentially altering migratory properties. Interestingly, previous reports have found that ablation of Rho‐signaling by a RhoA‐GTPase‐KO in microglia triggers spontaneous microglial activation, as shown by increased TNF expression and secretion (Socodato et al. 2020).

In addition to the canonical pathway analysis, we also analyzed the interplay of the differentially regulated proteins and the predicted outcome for the organism (Figure 1e, Figure S8). The disease/function network analysis is based on the entire proteomic profile as revealed in the analysis, which showed that dysregulated DEPs in *Cx3cr1* ^*−/−*^ mice were strongly connected with predicted elevation of motor dysfunction, apoptosis, and movement disorders. This finding is in line with previous results, in which both *Cx3cr1* ^*+/−*^ and *Cx3cr1* ^*−/−*^ mice display motor learning and structural neuroplasticity deficits (Gellner et al. 2024). As we found a down‐regulation in Rho‐signaling, likely related to microglia, we assumed a change in microglial functionality resulting in changes in their mobility. Thus, we conducted further functional analysis in the murine cortex to assess how specifically microglia migration is affected by the different *Cx3cr1* genotypes.

### 2.2 CX3CR1 Modulates Migration and Process Extension of Microglia

**FIGURE 2:**
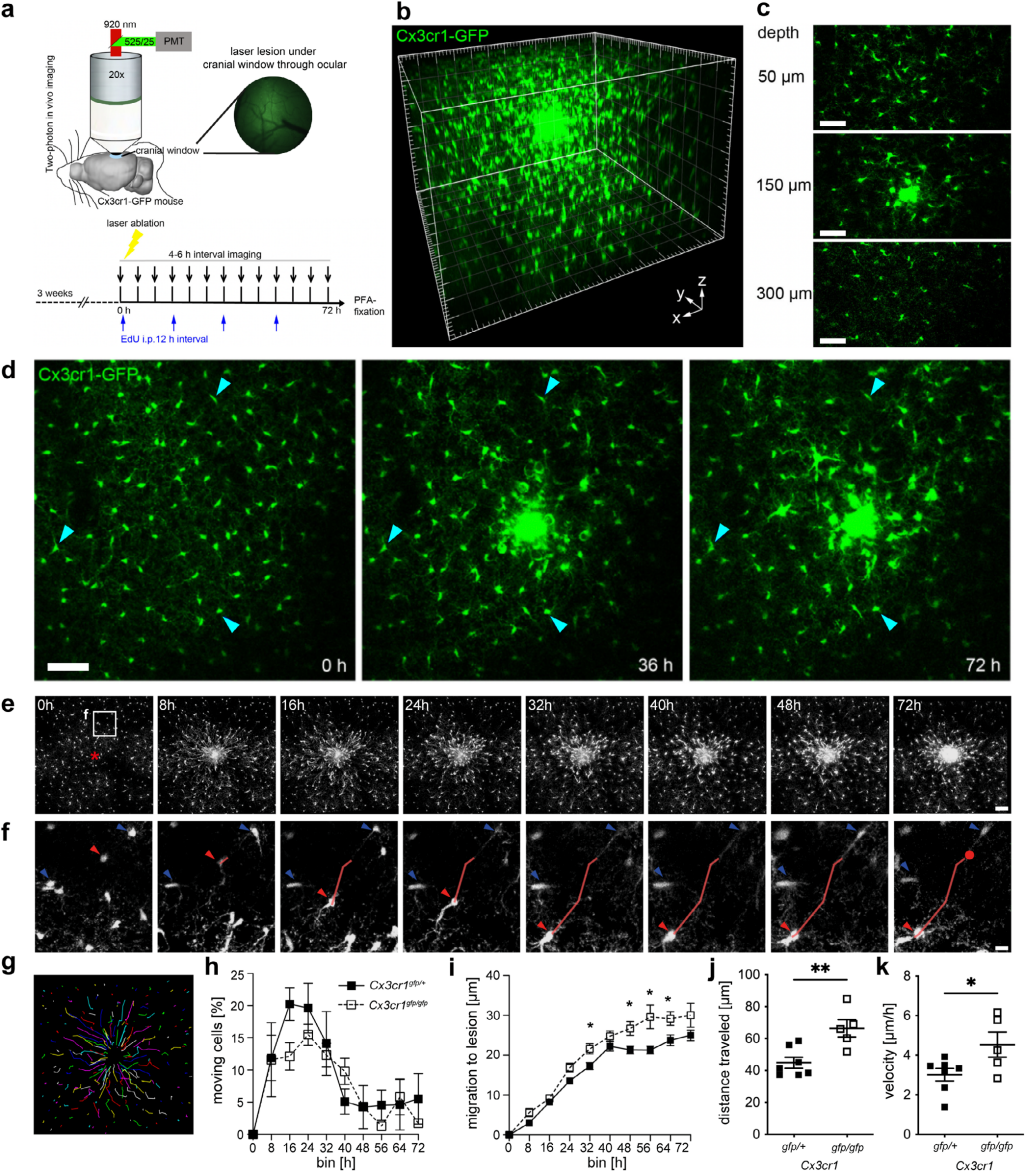
CX3CR1 modulates microglia migration toward a laser lesion. (a) Schematic of a mouse fixed under a two‐photon laser scanning microscope and the timeline of the experiments. Mice used for imaging of microglia migration underwent cranial window surgery over the right somatosensory cortex to install a circular coverslip (∅5 mm) as described. (b) Exemplary 3‐dimensional reconstruction of a 300 μm z‐stack showing microglia (green) around the lesion. Major tick = 50 μm (c) Optical slices from various depths (50–300 μm) illustrating microglia accumulation around the lesion. Scale bars = 50 μm. (d) Maximum intensity projection (60 μm) of a laser lesion illustrating three time‐points before and after the lesion. Cyan arrowheads indicate non‐moving microglia. Scale bar: 50 μm. (e) Time‐lapse two‐photon in vivo images of cortical microglia before and up to 72 h after laser lesion induction. The red star marks the lesion's position. As described in the methods section, twenty slices spanning 60 μm and containing the lesion are displayed. Scale bar = 75 μm (f) Zoom‐in from white inset in (e) illustrating a migrating microglial cell (red arrow) and non‐moving microglia (blue arrowheads) over the 72 h imaging period. Cell movement is tracked over the imaging period (red line) with the red dot representing the starting point. Scale bar = 15 μm (g) Color‐coded tracks of migrating and sessile microglia in (e). (f,g) Exemplary images were taken from *Cx3cr1* ^*gfp/+*^ mice. (h) The fraction of migrating microglia over time of *Cx3cr1* ^*gfp/+*^ versus *Cx3cr1* ^*gfp/gfp*^ is not different between the two genotypes (*p* > 0.05; determined using Two‐way ANOVA followed by Šídák's multiple comparisons test). (i) Distance traveled toward the lesion over time is significantly increased in *Cx3cr1* ^*gfp/gfp*^ compared to *Cx3cr1* ^*gfp/*+^ mice (*p < 0.05; determined using Two‐way ANOVA followed by Šídák's multiple comparisons test). (j) Overall distance of microglia traveled toward the lesion is significantly increased in *Cx3cr1* ^*gfp/gfp*^ mice. (*n* = 351 cells in *Cx3cr1* ^*gfp/gfp*^ and 159 cells in *Cx3cr1* ^*gfp/+*^ mice *Cx3cr1* ^*gfp/+*^: 44.9 ± 3.4 μm; *Cx3cr1* ^*gfp/gfp*^: 66.4 ± 5.5 μm; **p < 0.01; determined using an unpaired *t*‐test) (k) Microglia migration velocity is significantly increased in *Cx3cr1* ^*gfp/gfp*^ in comparison to *Cx3cr1* ^*gfp/+*^ mice (*n* = 719 cells in *Cx3cr1* ^*gfp/gfp*^ and 461 cells in *Cx3cr1* ^*gfp/+*^ mice *Cx3cr1* ^*gfp/+*^: 3.1 ± 0.3 μm/h; *Cx3cr1* ^*gfp/gfp*^: 4.5 ± 0.6 μm/h; *p < 0.05; determined using an unpaired *t*‐test). (h–k) *N* = 7 *Cx3cr1* ^*gfp*/+^ (6 males, 1 female) *N* = 5 *Cx3cr1* ^*gfp*/*gfp*^ (3 males, 2 females) mice.

To investigate the migration of microglia around a laser lesion in the brain in vivo, we carried out repetitive two‐photon in vivo imaging of individual GFP‐expressing microglia over a period of 72 h with 4–6‐h time intervals (Figure 2a, Video S1). *Cx3cr1* ^*gfp*^ mice were used. Green fluorescent protein (GFP) is inserted into the chemokine receptor *Cx3cr1*‐locus, leading to *Cx3cr1*‐deficiency in homozygous *Cx3cr1* ^*gfp/gfp*^ mice. As controls, heterozygous *Cx3cr1* ^*gfp/+*^ mice were used as they still feature labeled microglia while maintaining one allele of *Cx3cr1*. Microglial GFP‐expression labels the microglia for in vivo microscopy and imaging of the microglia movements (Figure 2b–d).

Initially, we investigated microglial cell body migration starting 3–4 h after laser lesion induction (Figure 2e,f). We tracked on average 166 ± 10 microglia per mouse and lesion (Figure 2g). Most cells in the imaged field of view (74.0% ± 3.5%) remained at their initial position in the brain parenchyma, while a substantial proportion of 26.0% ± 3.5% started to migrate. The fraction of migrating cells, however, was not constant over time; it increased rapidly toward a peak (at 21 ± 3 h after the laser lesion) and declined to 4.3% ± 0.8% moving cells after 48 h (Figure 2h). Time to peak was not different between *Cx3cr1* ^*gfp/+*^ and *Cx3cr1* ^*gfp/gfp*^ mice (Figure 2h). Surprisingly, the distance traveled toward the lesion was significantly increased in *Cx3cr1* ^*gfp/gfp*^ compared to *Cx3cr1* ^*gfp/+*^ microglia (Figure 2i,j). Additionally, *Cx3cr1* ^*gfp/gfp*^ microglia exhibited a significant 41% increase in migration velocity in comparison to *Cx3cr1* ^*gfp/+*^ microglia (Figure 2k). The lesion diameter was the same in both experimental groups, ruling out any biasing effect due to different lesion size in chemotactic gradient (Figure S2a). Simultaneously, the recruitment radius in which microglial movements are observed, and microglial density remained unchanged between the two conditions (Figure S2b,d,e). Moreover, MI and the total percentage of migrating microglia were unaffected by genotype, indicating that *Cx3cr1* ^*gfp/gfp*^ microglia were able to migrate, though they migrated further at a higher speed (Figure S2c,f). Taken together, migration distance and velocity of microglia are enhanced in *Cx3cr1* ^*gfp/gfp*^ microglia, indicating a role of CX3CR1 in regulating microglial dynamics in response to laser‐induced cortical injury.

**FIGURE 3:**
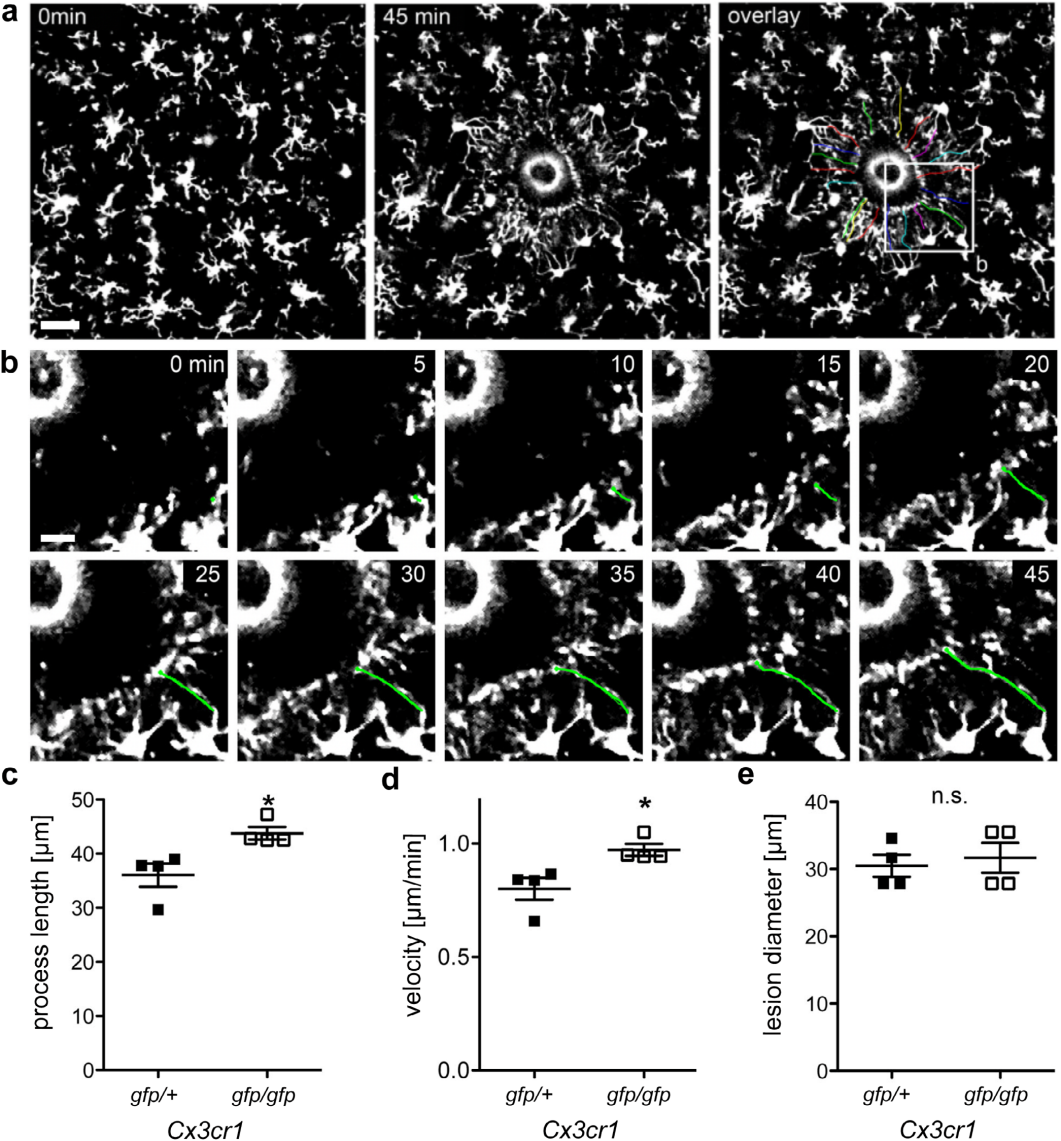
Immediate extension of microglia processes in response to a laser lesion is modulated by CX3CR1. (a) Exemplary overview z‐plane of microglia before (0 min) and after a laser lesion (45 min). The third panel illustrates individual, tracked processes extending toward the lesion 45 min after lesion induction (color‐coded lines). Scale bar = 25 μm (b) Zoom‐Ins of the marked area in (a) depicting an individually‐tracked microglial process (green) over the 45 min recording periods in 5 min intervals. Scale bar = 10 μm. Exemplary images were taken from *Cx3cr1* ^*gfp/+*^ mice. (c, d) The fine processes length (c) and average velocity (d) of microglia fine processes extending toward the lesion comparing *Cx3cr1* ^*gfp/gfp*^ mice (fine process length: *Cx3cr1* ^*gfp/+*^: 36.0 ± 2.1 μm; *Cx3cr1* ^*gfp/gfp*^: 43.8 ± 1.2 μm; *n* = 4 mice/group; *p < 0.05; velocity: *Cx3cr1* ^*gfp/+*^: 0.80 ± 0.05 μm/min; *Cx3cr1* ^*gfp/gfp*^: 0.97 ± 0.03 μm/min). (e) Comparison of the lesion diameter between *Cx3cr1* ^*gfp/+*^ and *Cx3cr1* ^*gfp/gfp*^ mice (lesion diameter: *Cx3cr* ^*gfp/+*^: 31 ± 2 μm; *Cx3cr1* ^*gfp/gfp*^: 32 ± 2 μm; *n* = 4 mice/group; *p* > 0.05). Data in (c, d and e) are displayed over mice (*n* = 4 mice/group; 3 males, one female per group), depicting the mean ± SEM.

During the first hours after laser lesion, migrating microglia change their morphology. They form a leading process while simultaneously retracting the other processes (Figure S4a,b). Contrastingly, sessile cells retain their branched morphology, and don't form a leading process (Figure S4c). The length of the leading process 12 h after lesion induction is not different compared to *Cx3cr1* ^*gfp/+*^ and *Cx3cr1* ^*gfp/gfp*^ mice (Figure S4d). In addition to microglial cell body migration, we analyzed the effect of *Cx3cr1*‐deficiency on the kinetics of microglia processes' extension toward a laser lesion. We repetitively imaged in 5‐min time intervals throughout the first hour after lesion induction (Figure 3a,b; Video S2). Microglial fine processes extended toward the lesion earlier than the cell body (Figures 2e, 3a,b), indicating that process extension precedes cell body migration. Interestingly, we identified decreased microglial area coverage in *Cx3cr1* ^*gfp/gfp*^ mice measured by fluorescent immunohistochemistry with Iba1 antibodies. P2Y12R microglial area coverage remained unchanged, whereas P2Y12R signal normalized to Iba1 showed a slight but not significant increase in *Cx3cr1* ^*gfp/gfp*^ mice suggesting unchanged P2Y12 receptor expression in Cx3cr1‐deficient microglia (Figure S5a–d).

It is important to distinguish between microglia in proximity to the lesion and microglia in peripheral regions more distant from the lesion. To assess the immediate reaction of microglia at the lesion site, we repetitively imaged in 5‐min time intervals throughout the first hour after lesion induction (Figure 3a,b; Video S2). *Cx3cr1* ^*gfp/gfp*^ microglia around the lesion showed significantly longer processes, extending toward the lesion, than *Cx3cr1* ^*gfp/+*^ microglia (Figure 3c). Similarly, the velocity of process extension was significantly augmented (Figure 3d), whereas lesion diameter remained unaffected (Figure 3e). These initially responding microglia are not migrating long distances toward the lesion, but the augmented response of microglia completely lacking CX3CR1 is similar to the faster migration observed in more peripheral microglia.

Interestingly, microglia motility, the overall turnover rate of the microglia fine processes, remains unaffected in the absence of a laser‐lesion between the conditions, indicating that surveillance capacity, as well as non‐migration‐related functions, are independent of *Cx3cr1* (Figure S5). Taken together, these data underscore the importance of CX3CR1 in facilitating microglial dynamics in reaction to a cortical laser lesion.

### 2.3 Resident Microglia Migrate to the Lesion and Proliferate

**FIGURE 4:**
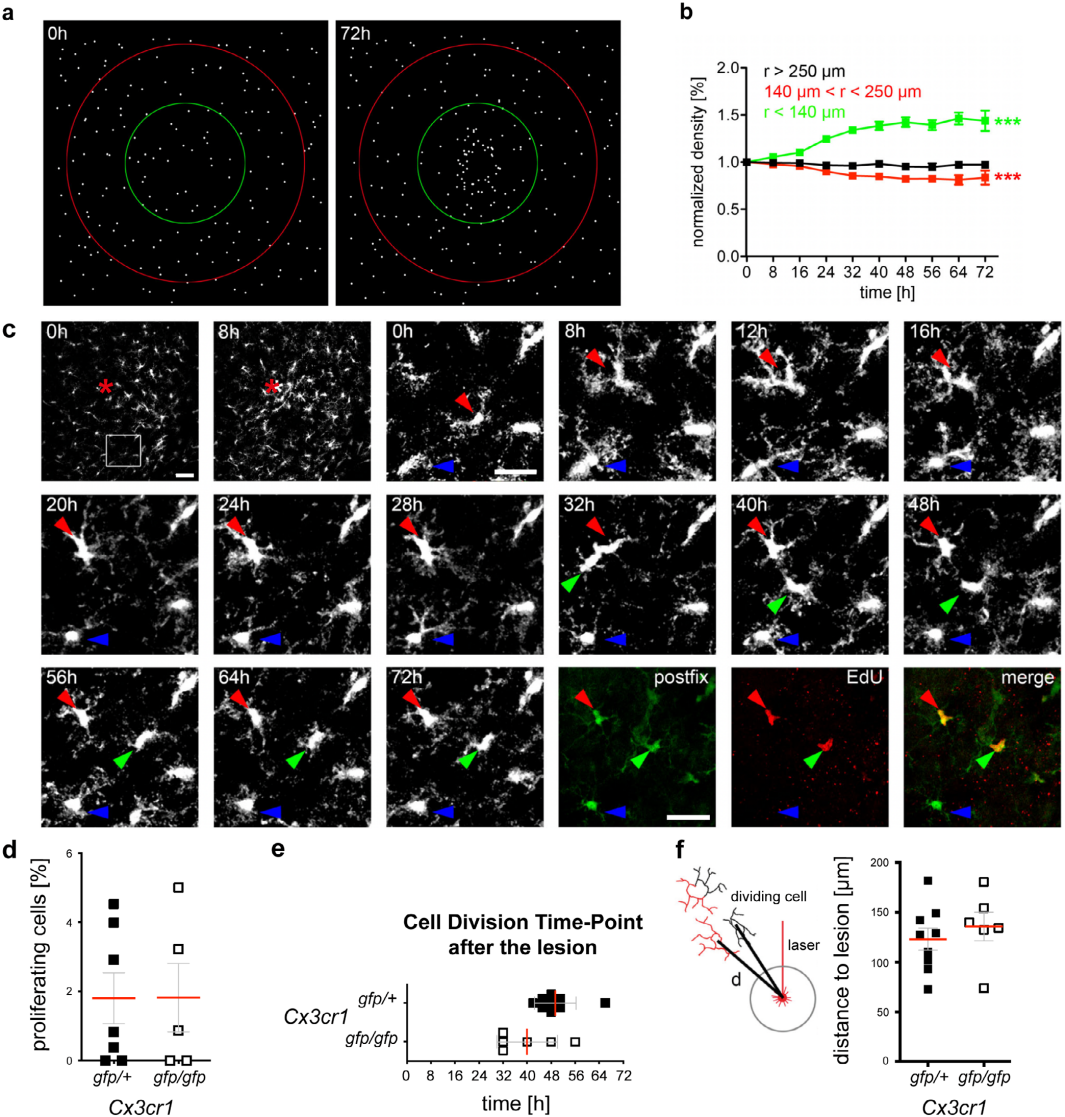
Microglia migration and proliferation events of resident microglial populations. (a) Recording microglia density changes around the lesion: The microglia distribution (each dot represents a tracked microglia) in the same mouse at the same cortical position before (0 h) and 72 h (72 h) after the laser lesion is shown. Red circle: Radius of 250 μm, green circle: Radius of 140 μm around the lesion. Star: Lesion (b) Changes in microglia density [%] over time, normalized on the TP0 before lesion induction. The density of microglia around the lesion (*r* < 140 μm) increases significantly, while the density in the region between 140 and 250 μm radius decreases significantly. The microglia density beyond (*r* > 250 μm) stays stable over time (*n* = 11 mice; ***p < 0.001; determined using Two‐Way ANOVA). (c) Imaging of microglia proliferation after lesion induction and post hoc identification of proliferation using the marker EdU in immediately perfused animals. Overviews (first two panels) of a lesion, where a division event was observed. The Zoom‐Ins show a proliferating cell during in vivo imaging (panels 2–15) and after immediate perfusion and relocation (panels 16–18). Arrows: Dividing microglia (red, green), non‐dividing microglia (blue). Scale bars: 50 μm (Overviews), 25 μm (Zoom‐ins). (d) The fraction of proliferating microglia averaged over mice was independent of *Cx3cr1*‐deficiency (*Cx3cr1* ^*gfp/+*^: 1.8% ± 0.7%; *Cx3cr1* ^*gfp/gfp*^: 1.8% ± 1.0%; *p* > 0.05; determined using an unpaired *t*‐test, *n* = 5 *Cx3cr1* ^*gfp/gfp*^ and 7 *Cx3cr1* ^*gfp/+*^ mice). (e) Microglia proliferation events took place about 40 h after induction of the laser lesion and independent of *Cx3cr1* (*Cx3cr1* ^*gfp/+*^: 49 ± 2 h; *Cx3cr1* ^*gfpgfp*^: 40 ± 4 h; *p* > 0.05; determined using an unpaired *t*‐test). (f) Before division, microglia migrate toward the lesion and proliferate in its proximity. Proliferation unaffected by *Cx3cr1* knockout (*Cx3cr1* ^*gfp/+*^: 123 ± 11 μm; *Cx3cr1* ^*gfp/gfp*^: 136 ± 14 μm; *p* > 0.05; determined using an unpaired *t*‐test). (e–f) *n* = 9 events in 7 *Cx3cr1* ^*gfp/+*^ mice vs. *n* = 6 events in 5 *Cx3cr1* ^*gfp/gfp*^ mice.

We observed an increase in microglia density in proximity to the lesion (*r* < 140 μm), a decrease within a radius of 140 to 250 μm, and a constant density more than 250 μm away from the lesion (Figure 4a,b). To decipher the origin of microglia in proximity to the lesion, we followed individual microglia over time up to 72 h with 4–6 h acquisition intervals (Video S1). Strikingly, our observations revealed that the majority (~98%) of microglia accumulating around the lesion were previously sessile in the brain parenchyma and started to migrate toward the lesion (Figure 2h). We occasionally observed newly appearing cells in proximity to a neighboring microglial cell (Figure 4c; Video S3). To identify if these microglia originated from cell division, we injected the proliferation marker 5‐ethynyl‐2′‐deoxyuridine (EdU) during the 72h in vivo imaging period (Figure 2a). After imaging, we retrieved putative dividing microglia from PFA‐fixed brain slices and demonstrated the division by EdU staining. As visualized, cell division was in some cases preceded by an increase in microglial soma volume (Figure 4c). We analyzed the spatio‐temporal distribution of dividing microglia dependent on *Cx3cr1*‐deficiency. In comparison to the fraction of migrating microglia (26.0% ± 3.5%), we only detected about 1.8% dividing microglia that contributed to local cell accumulation around the laser lesion (Figure 4d). Divisions took place 49 ± 2 h in *Cx3cr1* ^*gfp/+*^ compared to 40 ± 4 h in *Cx3cr1* ^*gfp/gfp*^ mice at a time when the peak of migration had already been reached (Figure 4e). In addition, division close to the lesion is independent of CX3CR1 (Figure 4f). Interestingly, cell proliferation started after microglia processes extension and migration to the lesion (Figure 4e). Proliferation is therefore a subsequent response upon a laser‐induced brain parenchyma injury. Concluding, the observed migrating microglia are initially static and resident in the brain parenchyma. They start moving about 8 h after lesion induction. After migration toward the lesion, a small fraction of microglia proliferates in proximity to the lesion. Local proliferation is independent of CX3CR1.

### 2.4 Accumulating Microglia in the First 72 h After Lesion Induction Do Not Originate From Infiltrating Monocytes

**FIGURE 5:**
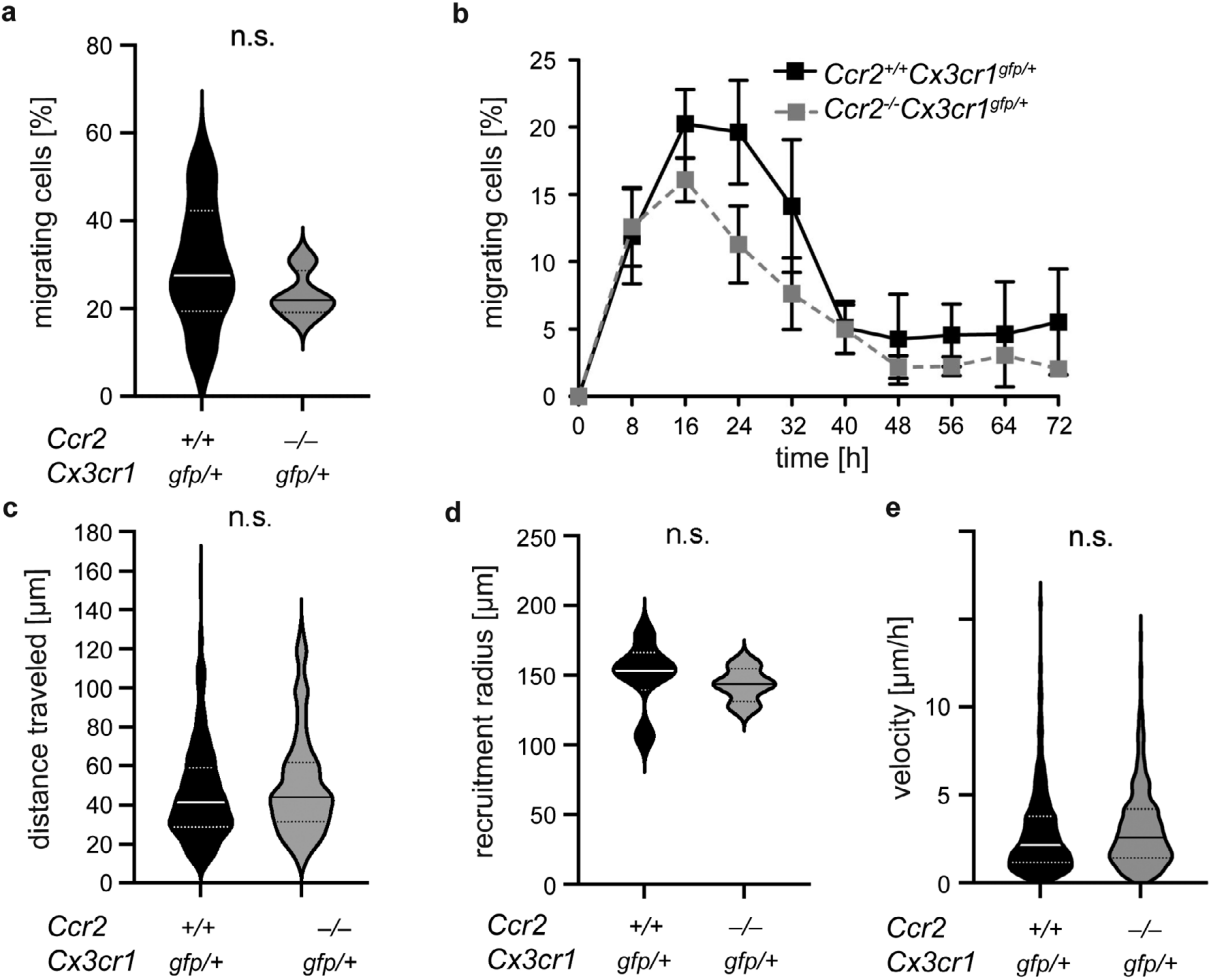
Peripheral monocytes do not contribute to microglia accumulation around the lesion. (a, b) In vivo imaging to exclude a contribution of peripheral monocyte populations to the cells accumulating around the lesion was conducted as described previously. The fraction of migrating microglia cells overall (a) and over time (b) was not different comparing *Ccr2* ^*+/+*^ *Cx3cr1* ^*gfp/+*^ and *Ccr2* ^*−/–*^ *Cx3cr1* ^*gfp/+*^ mice (*Ccr2* ^*+/+*^ *Cx3cr1* ^*gfp/+*^: 29.8% ± 5.8% versus *Ccr2* ^*−/–*^ *Cx3cr1* ^*gfp/+*^: 23.2% ± 2.7%; *n* = 4–6 mice/group; *p* > 0.05; Two‐way ANOVA followed by Šídák's multiple comparisons test). (c) The traveled distance of microglia is unaffected by *Ccr2*‐deficiency (*Ccr2* ^*+/+*^ *Cx3cr1* ^*gfp/+*^: 42.7 ± 2.0 μm; *Ccr2* ^−/–^ *Cx3cr1* ^*gfp/+*^: 43.9 ± 2.1 μm; *p* > 0.05; *n* = 167 cells from *Ccr2* ^*−/–*^ *Cx3cr1* ^*gfp/+*^ and 351 cells from *Ccr2* ^*+/+*^ *Cx3cr1* ^*gfp/+*^mice; determined using an unpaired *t*‐test). (d) The average recruitment radius was independent of *Ccr2* (*Ccr2* ^*+/+*^ *Cx3cr1* ^*gfp/+*^: 151 ± 10 μm; *Ccr2* ^*−/–*^ *Cx3cr1* ^*gfp/+*^: 143 ± 6 μm; *p* > 0.05; determined using an unpaired *t*‐test). (e) Microglia velocity was independent of *Ccr2* knockout (*Ccr2* ^*+/+*^ *Cx3cr1* ^*gfp/+*^: 3.2 ± 0.1 μm/h; *Ccr2* ^*−/–*^ *Cx3cr1* ^*gfp/+*^: 3.4 ± 0.1 μm/h; *p* > 0.05; *n* = 719 cells from *Ccr2* ^*−/–*^ *Cx3cr1* ^*gfp/+*^ and 405 cells from *Ccr2* ^*+/+*^ *Cx3cr1* ^*gfp/+*^mice; determined using an unpaired *t*‐test). *N* = 4 *Ccr2* ^*−/–*^ *Cx3cr1* ^*gfp/+*^ and 4 *Ccr2* ^*+/+*^ *Cx3cr1* ^*gfp/+*^mice. Note that the *Ccr2* ^*+/+*^ control conditions are the same measurements as the *Cx3cr1* ^*gfp/+*^ values in Figure 1.

Transient recruitment of *Cx3cr1*‐expressing inflammatory monocytes from the blood has been shown to depend on the chemokine receptor 2 (CCR2) expression in a model of experimental autoimmune encephalitis (EAE) (Ajami et al. 2011). Furthermore, monocyte‐derived cells have been found to enter the brain under certain circumstances (Frumer et al. 2024). Our results of resident microglia migrating and accumulating at the lesion site indicate that the contribution of blood‐derived monocytes might be limited. To rule out that *Cx3cr1*‐positive monocytes contributed to the accumulating cells at the lesion, we used *Ccr2*‐KO mice, where monocyte recruitment from blood is prevented. Therefore, we crossbred *Ccr2*‐deficient mice with *Cx3cr1* ^*gfp*^ mice to yield *Cx3cr1* ^*gfp/+*^ *Ccr2* ^−/−^ mice and carried out a laser lesion experiment as described before. The fraction of migrating microglia in *Cx3cr1* ^*gfp/+*^ *Ccr2* ^*+/+*^ mice changes similarly over the imaging time period with a peak of microglia migration at 16 h post lesion induction (Figure 5b). We did not detect any significant differences in the proportion of migrating cells comparing *Cx3cr1* ^*gfp/+*^ *Ccr2* ^*+/+*^ and *Cx3cr1* ^*gfp/+*^ *Ccr2* ^−/−^ (Figure 5a). Moreover, kinetics, distance traveled, recruitment radius, and velocity remain unchanged between the conditions (Figure 5c–e). These results suggest that infiltrating monocyte precursors of macrophages do not seem to contribute to the accumulation of cells around the laser lesion within the first 3 days after lesion induction. In addition, these data indicate that microglial chemotaxis toward the laser lesion is independent of the monocyte chemoattractant protein‐1 (MCP1), the ligand for CCR2.

## 3 Discussion

So far, the influence of CX3CR1 on long‐term microglia migration has not been studied in detail. Quantitative global proteomic analysis suggested alterations in migration‐relevant signaling pathways of cortical cells. Hence, we performed in vivo two‐photon imaging to characterize the role of CX3CR1 in microglia migration, one elementary feature of those immune cells. In prior studies, migration and relevant factors regulating migration were mostly studied in vitro, and only a few studies focused on microglial migration in vivo. Specifically, the dynamics of the microglial response to cortical laser lesions remained elusive. Our in vivo data demonstrate that CX3CR1 modulates microglial migration toward a laser lesion. Specifically, the absence of CX3CR1 led to increased microglial velocity and migration distance toward the lesion, accompanied by enhanced extension of fine processes. We also observed local proliferation of microglia accumulating around the lesion (~2% of imaged cells). Using *Ccr2*‐knockout mice, we excluded the contribution from peripheral monocyte‐derived cells.

By analyzing the proteome of the cortex, we identified a variety of pathways that are differentially regulated in the absence of CX3CR1. Although the proteomics results cannot provide information about individual cell types, the results are likely to reflect microglial changes because CX3CR1 is primarily expressed by microglia in the cortex. Interestingly, among the identified DEPs, many are related to the cytoskeleton and migration. This analysis reveals a novel functional link between CX3CR1 and the cytoskeleton, as well as cellular movement and migration. Most prominently, the IPA results from proteomic analysis imply a downregulation of RHO‐signaling in CX3CR1‐deficient microglia and cortical macrophages. This is accompanied by a downregulation of the associated signaling pathways regarding the cytoskeleton and migratory capacity of cells within the cortical tissue of *Cx3cr1*‐knockout mice. Our in vivo obtained results support previous in vitro results from a variety of cell culture and cancer models, where it has been established that CX3CR1/CX3CL1 signaling interacts with RHO‐signaling pathways (Zeng et al. 2023; Zheng et al. 2022; Liu et al. 2024). We could show a downregulation of Rho‐signaling in the absence of CX3CR1 in *Cx3cr1* ^*−/−*^ compared to *Cx3cr1* ^*+/−*^ mice, which is in line with prior results that conclude an increase in Rho signaling upon increasing CX3CR1 signaling. Initially, this result seemed counterintuitive considering our results of enhanced migration and fine processes extension. However, it has been shown that RhoA‐GTPase deficiency in Cx3cr1^+^ cells, resulting in ablation of Rho‐signaling, is associated with spontaneous microglia activation and increased expression of pro‐inflammatory cytokines in vivo (Socodato et al. 2020). Additional in vitro results confirmed that a tight control of RhoA activity is necessary to control the microglial inflammatory response since a complete Rho‐KO led to microglial cell death, whereas a slight reduction led to increased inflammatory activity. In contrast, sustained RhoA signaling inhibited inflammatory microglial activity (Socodato et al. 2023). These results imply that a downregulation, but not total deactivation, of Rho‐signaling induces a microglial state shift, which would make them more sensitive to an injury such as a laser lesion. Thus, our finding of reduced RhoA‐signaling may be associated with a microglial state shift, rendering them alert for a faster injury response. Interestingly, our microglial motility data do not imply a higher baseline inflammatory activity of Cx3cr1^gfp/gfp^ microglia in the absence of an activating stimulus such as lesion induction. Another discovery is that the profile of altered DEPs predicted elevated motor dysfunction and movement disorders in *Cx3cr1‐KO* mice. Interestingly, our proteomics‐based predictions concerning motor dysfunction have already been described, comparing the motor behavior of *Cx3cr1* ^*+/−*^ with *Cx3cr1* ^*−/−*^ mice (Gellner et al. 2024; Pei et al. 2024).

The enrichment of cytoskeletal regulation and neuronal networks (e.g., ‘motor dysfunction’ in Figure 1e) (Gyoneva et al. 2019; Zhang et al. 2014; Butovsky et al. 2014; Hickman et al. 2013) also suggests that microglial alterations may secondarily influence the proteome of neighboring neurons or astrocytes—a signal that is lost in sorted‐cell transcriptomics but captured in bulk proteomics (Gyoneva et al. 2019; Zhang et al. 2014; Butovsky et al. 2014; Hickman et al. 2013). Microglial function relies on their motility and ability to migrate. By extending and retracting their processes as well as active migration toward sites of injury within the CNS, microglia exert several functions. We demonstrate that deficiency of the chemokine receptor CX3CR1 led to an increased migration velocity and traveled distance of microglia toward a laser lesion compared to *Cx3cr1*‐haploinsufficiency. These findings are in accordance with previous research stating that under healthy conditions, a small amount of microglia is mobile. Focal vascular injury induces migration toward the site of injury in a sex‐specific manner (Boghozian et al. 2023; Eyo et al. 2018). Interestingly, a previous study in the primary visual cortex found a similarly amplified microglial process response to laser ablation in *Cx3cr1* ^*null*^ (*Cx3cr1* ^*gfp/gfp*^ and *Cx3cr1* ^*gfp/−*^) mice compared to heterozygous *Cx3cr1* ^*gfp/+*^ mice (Lowery et al. 2017). Therefore, our finding of increased microglial process extension in *Cx3cr1*‐deficient mice is in line with previous literature. The complete loss of Cx3cr1 seems to render microglia more reactive toward a pathological lesion model, which, next to the imaging data, can also be concluded from our proteomics data, suggesting a down‐regulation in Rho‐signaling and thus an increase in alertness. Additionally, previous work reported unaltered microglial density, morphology, and, like our data, also motility in heterozygous and homozygous *Cx3cr1* ^*gfp*^ mice (Lowery et al. 2017). Interestingly, a study conducted in retinal explants from homo‐ and heterozygous *Cx3cr1* ^*gfp*^ mice found opposite results in terms of the microglial processes' response toward a laser‐induced injury and migratory patterns toward this lesion (Liang et al. 2009). Cell‐culture based models showed that upregulation of CX3CR1 promotes microglia migration in ischemic‐stroke glucose‐deprived conditions (Cao et al. 2019). Notably, microglial behavior in these models is not directly comparable to our study and therefore differs from our results. We therefore suggest a context‐dependent regulation of microglia migration.

Fast and directed migration is accompanied by a change in microglial morphology, including retraction of most fine processes and formation of a leading process. This change in microglial polarization occurs within the first hours of pathology sensing and has been described before (Smolders et al. 2019) as one crucial step of directed microglia movements through complex tissues, such as the brain.

The timing of the transition from sessile to migration toward a brain lesion is critical. Microglia need time to initially sense and subsequently react to a brain lesion. We observed increased velocity of *Cx3cr1* ^*gfp/gfp*^ microglial fine processes in the first hour after lesion induction compared to *Cx3cr1* ^*gfp/+*^ microglia. These are microglia that are closest to the lesion. However, these cells do not migrate over long distances compared to microglia at more remote positions that start to migrate approximately 8 h after lesion induction. During migration initiation, microglia form one long, leading process, which is different from the processes initially (first 1 h) extended toward the lesion by microglia close to the lesion. The length of movement initiation processes of microglia at remote locations was not different at 12 h after lesion, comparing *Cx3cr1* ^*+/−*^ with *Cx3cr1* ^*−/−*^ mice. These findings suggest that the increased traveled distance by *Cx3cr1* ^*−/−*^ microglia is not based on a faster migration initiation.

We further found an increase in microglial density ~140 μm around the lesion. Similar patterns of spatial microglial migration were found in the same mouse model upon microhemorrhage induction (Ahn et al. 2018). Similarly, seizures as well as whisker trimming were found to induce microglia migration (Eyo et al. 2018). These studies utilized the same *Cx3cr1* ^*gfp/+*^ mice and clearly showed the onset of microglia migration upon different stimuli; however, there is a lack of investigation of CX3CR1‐dependency. Thus, we reveal a novel role of CX3CR1 in regulating characteristics of microglial migration in vivo. In a similar manner, but on a smaller timescale, extension and velocity toward a lesion are affected by CX3CR1‐deficiency, which underlines the role of CX3CR1 in the fine‐tuning of microglial motion in response to brain damage. However, under healthy conditions, our and a previous study found unchanged microglial motility comparing *Cx3cr1* ^*+/−*^ *and Cx3cr1* ^*−/−*^ *knockout mice* (Fuhrmann et al. 2010), *indicating that microglial processes are differentially regulated under unperturbed and injury conditions. In line with this, a* highly directional process extension of microglia toward ATP release, because of brain tissue damage, has been described in vivo (Davalos et al. 2005). The link between ATP and microglia movement was further underlined in a study investigating the effect of the P2Y12‐receptor knockout, which found that in these mice, microglial rearrangement is significantly reduced (Eyo et al. 2018). We neither found P2Y12 receptor expression changes in our proteomics data set, nor in our immunohistochemical analyses for P2Y12 receptor. These data suggest that CX3CR1, independently of P2Y12 receptor, modulates migration toward a laser lesion.

In our model, *Ccr2 deficiency* did not affect microglia migration. In addition, similar numbers of accumulating cells at the lesion in *Ccr2‐*deficient mice exclude a contribution of CX3CR1^+^CCR2^+^ monocytes from the blood. The *Ccr2 independence* of microglial migration also implies that its ligand MCP‐1 does not play a major role in regulating microglial migration in the context of brain lesions.

Occasionally, we observed newly appearing cells around the lesion due to local proliferation. Proliferation of microglia has been proposed and described over longer observation periods, in which fluorescently labeled microglia were imaged over the course of many weeks. Putative division events around amyloid lesions, similarly to what we found, were sometimes preceded by an increase in soma volume (Füger et al. 2017). However, the fusion or simple neighboring of two microglia might easily be misinterpreted as a division event. Therefore, definite proof of divisions observed with imaging methods requires either higher spatio‐temporal resolution or the combination of imaging with DNA‐intercalating markers like EdU and *post‐mortem* retrieval of the same cells using immunohistochemistry. Here, we were able to prove local microglia proliferation after migration toward a laser lesion using EdU as a proliferation marker. Microglia proliferation around an infarct in the brain parenchyma was also shown in prior studies, using EdU as a proliferation marker (Ahn et al. 2018). Therefore, proliferation to a much smaller extent than migration contributes to local accumulation around a laser lesion. After EdU‐injection, we did not identify proliferative events on the contra‐lateral hemisphere and thus hypothesize that the proliferative events around the lesion were caused by the cortical lesion itself. The published value for proliferating microglia in the murine cortex is approximately 0.5% (Askew et al. 2017), which is lower than the proliferation rate of ca. 1.8% that we found in both genotypes following lesion induction. Therefore, we assume that microglia proliferation is increased by our laser‐induced brain injury.

**FIGURE 6:**
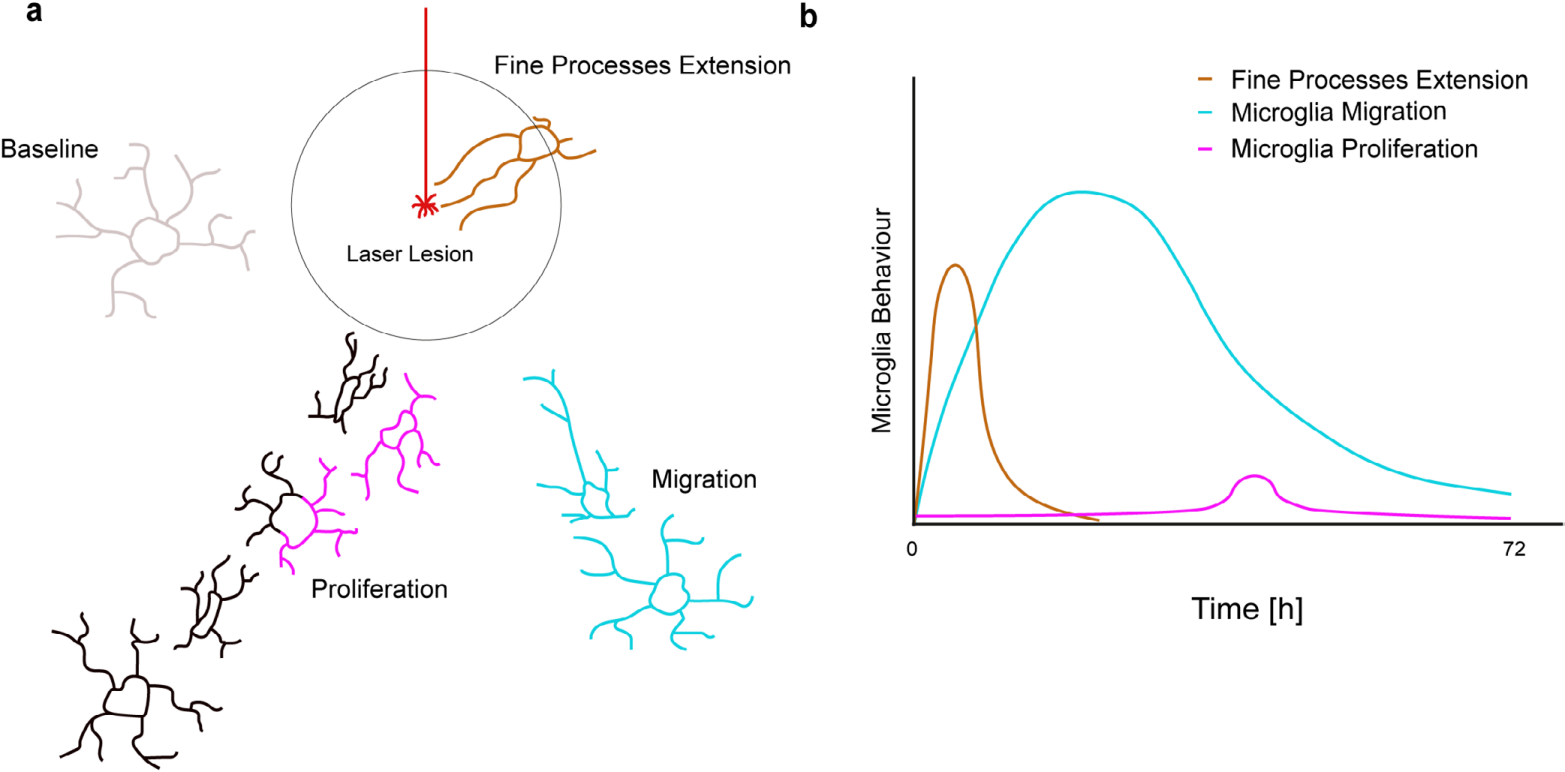
Schematic of microglia expansion kinetics. (a) Under baseline conditions (gray), microglia remain at their position and are constantly screening the brain parenchyma by protracting and retracting their fine processes. Upon induction of a laser lesion, microglia in proximity to the lesion are the first responders and extend fine processes toward the lesion within minutes (brown). This process's extension is regulated via CX3CR1, as a complete loss leads to an increased extension of the initial processes toward the lesion. Subsequently, after about 12 h, about 25% of resident microglia in the brain parenchyma start to migrate toward the lesion (blue) after forming a long leading process toward the lesion. In addition to migration, an even lower fraction, of about 2% of the imaged microglia within the FOV migrates and proliferates in proximity to the lesion (black and magenta). (b) Proposed model of the reaction of microglia toward a laser lesion in the cortex, starting at time‐point 0 (lesion‐induction) till 72 h‐post lesion.

Based on our findings, we propose a model for microglial kinetics upon laser lesion induction: Within the first hour after a laser lesion, microglial processes extend toward the lesion, which is followed by a highly directed migration toward the lesion. Migration peaks at approximately 16–24 h post‐lesion induction and, lastly, approximately 48 h after lesion induction, a small fraction of the microglia that migrated toward the lesion starts to proliferate there (Figure 6). Our findings are in line with previous results, which propose a similar cascade of events during microglia migration (Smolders et al. 2019).

Whether microglial state shift in response to a laser lesion is beneficial for tissue repair or if it hinders healing is still not fully elucidated. It is known that microglia are key neuroprotective factors after spinal cord injury, forming a glial scar (Bellver‐Landete et al. 2019) and exerting neuroprotective functions, as their depletion leads to a severely increased infarct size (Szalay et al. 2016; Marino Lee et al. 2021; Zhou et al. 2023). Contrastingly, however, growing glial scars were also found to impair axonal regeneration (Li et al. 2022) and microglia release glutamate, which is a major excitotoxic factor (Umebayashi et al. 2014). Hence, microglia seem to have a dichotomous role in response to CNS injury. We found that microglial process extension and migratory response toward laser lesion‐induced damage is the immediate CX3CR1‐dependent response to injury. Microglia migration toward the site of injury is thus the first step of a complex process of the microglial injury response and precedes their effector functions. Therefore, modulating migration via CX3CR1 might represent a novel strategy for the treatment of diseases, including a sterile innate immune response.

### 3.1 Limitations of the Study

Here we present data about CX3CR1‐dependent regulation of microglial migration and processes extension toward a laser lesion. We show that accumulation of microglia in our laser‐induced lesion model is modulated by CX3CR1; the functional consequences remain a topic for further follow‐up studies. Our experimental design, which involves 4‐h periodic imaging over 3 days, was intended to capture dynamic changes in microglial migration in response to injury. Nonetheless, we do not investigate long‐term microglia dynamics later than 3 days after lesion induction. Our study focuses on the extension of microglial processes toward the laser lesion and the regulation of migration dynamics by CX3CR1. However, once microglia arrive at the lesion, it is difficult to differentiate individual microglia, let alone their processes, due to extensive glial scarring and accumulation around the lesion. Therefore, it is very difficult to determine the fate of microglia as well as their leading processes once they are at the lesion. Microglial migration and processes extension/motility are complex biological processes that are best captured using in vivo microscopy following the changes over a long, yet unprecedented, period of time. Additionally, proteomics is employed to assess protein abundance. These methods—particularly imaging—provide descriptive insights into real‐time cellular changes. However, establishing definitive mechanistic links is difficult, as a detailed analysis of downstream CX3CR1‐signaling regulators is beyond the current scope of this work. The proteomics were performed in whole‐brain tissue, and we therefore cannot exclude that other cells than microglia contribute to the observed changes. Cx3cr1 in brain tissue is primarily expressed by microglia. We therefore believe that the observed changes are mainly driven by microglia.

## 4 Methods

### 4.1 Transgenic Mice

For label‐free proteomic analysis, *Cx3cr1* ^*CreERT2*^ mice that featured a homo‐. (*Cx3cr1* ^*−/−*^) or heterozygous (*Cx3cr1* ^*+/−*^) knockout of the fractalkine receptor, replaced with a CreERT2‐Recombinase were used (Yona et al. 2013). Animals were 14.6 ± 0.3 months old, and sexes were equally distributed within groups (Figure 1a). For the in vivo imaging experiments, *Cx3cr1* ^*gfp*^ mice at 5.4 ± 0.7 months. Both mouse lines were generously provided by Steffen Jung (Weizmann Institute of Science, Rehovot, Israel) (Jung et al. 2000) (Figure S7a). Genotyping of the *Cx3cr1* ^*CreERT2*^ mice was performed using the following primers: Forward: CAG TGA TGC TCT TGG GCT TCC, Reverse 1: TCA GTG TTT TCT CCC GCT TGC, Reverse 2: ATC AAC GTT TTG TTT TCG GA. *Cx3cr1* ^*−/−*^ yield one ca. 700 bp long fragment, *Cx3cr* ^*+/+*^yield one ca. 400 bp fragment, and *Cx3cr1* ^*+/−*^ yield two fragments of both sizes (Figure S7c).

Homozygous *Cx3cr1* ^*gfp/gfp*^ mice were crossed with heterozygous *Cx3cr1* ^*gfp/+*^ mice to yield homozygous and heterozygous littermates at a 1:1 ratio. The heterozygous *Cx3cr1* ^*gfp/+*^ mice retain a significant portion of the CX3CR1 receptor, while *Cx3cr1* ^*gfp/gfp*^ *mice* feature a complete *Cx3cr1* knockout (Figure S7a). Genotyping of the mice was performed using the following primers: IMR3945 5′‐TTCACG TTCGGTCTGGTGGG‐3′, IMR3946 5′‐GGTTCCTAGTGGAGCTAGGG‐3′, IMR3947 5′‐GAT CACTCTCGGCATGGACG‐3′. *Cx3cr1* ^*+/+*^ yields a single ~970 bp fragment, *Cx3cr1* ^*gfp/+*^ yields a ~1200 bp and a ~970 bp fragment, and *Cx3cr1* ^*gfp/gfp*^ a single ~1200 bp fragment. *Cx3cr1* ^*gfp/+*^ *Ccr2* ^*−/−*^ mice were generated by Daniel R. Engel and Christian Kurts by crossing *Cx3cr1* ^*+/+*^ *Ccr2* ^*−/−*^ with *Cx3cr1* ^*gfp/gfp*^ *Ccr2* ^*+/+*^ mice, yielding heterozygous *Cx3cr1* ^*gfp/+*^ *Ccr2* ^*+/−*^ offspring that were back‐crossed with *Cx3cr1* ^*+/+*^ *Ccr2* ^*−/−*^ mice (Kuziel et al. 1997) (Figure S7b). Since both *Cx3cr1* and *Ccr2* are located on the murine chromosome 9, we had to identify a crossing over event. *Cx3cr1* ^*gfp/+*^ *Ccr2* ^*−/−*^ mice were selected by levels of *Ccr2* and *Gfp* detected via Flow Cytometry of tail vein blood and genotyping using the following primers: P1: 5′‐TAA ACC TGG TCA CCA CAT GC‐3′; P2: 5′‐TCA CTT ACT TTA CAA CCC AAC C‐3′; P3: 5′‐GGA GTA GAG TGG AGG CAG GA‐3′; P4: 5′‐GAA GGC GAT AGA AGG CGA T‐3′. P1 and P2 amplify a 1.4 kb fragment of the wild‐type allele and a 3.2 kb fragment of the knock‐in allele, while P2 is located in a region that was not part of the original target allele, thereby checking for correct insertion. P1 and P3 amplify a 494 bp fragment of the wild‐type allele. P3 spans the BamH1 restriction site used for neomycin gene insertion. P1 and P4 amplify a 1.2 kb fragment, and P4 is located inside the neomycin gene (Figure S7d).

Mice were group‐housed in individually ventilated cages (IVC) under specific pathogen‐free (SPF) conditions with *ad libitum* access to food and water. Mice of both sexes were used. After surgery, the mice were separated and singly housed. All procedures were in accordance with an animal protocol approved by the DZNE and the government of North‐Rhine‐Westphalia (Az 84‐02.04.2017.A098).

### 4.2 Label‐Free Quantitative Proteomics Analysis

1 mg of mouse brain cortex from each genotype was dissected and processed using a modified filter‐aided sample preparation method as described (Scifo et al. 2015). The peptide samples were cleaned using C18‐reverse‐phase ZipTip (Millipore). Dried peptide digest was resuspended in 1% trifluoroacetic acid and sonicated in a water bath for 1 min before injection. Fractionated protein digests were analyzed in nano‐LC–Thermo Q Exactive Plus Orbi‐Trap Mass Spectrometer with parameters set as described (Gholizadeh et al. 2021). Following LC–MS/MS acquisition, the raw files were qualitatively analyzed by Proteome Discoverer, version 2.5 (Thermo Fisher Scientific). The identification of proteins by Proteome Discoverer was performed against the UniProt/SwissProt reviewed mouse protein database (release 10‐2022 with 17,139 entries) using the built‐in SEQUEST HT engine. The following parameters were used: 10 ppm and 0.02 Da tolerance for MS and MS/MS, respectively. Trypsin was used as the digesting enzyme, and two missed cleavages were allowed. The carbamidomethylation of cysteines was set as a fixed modification, whereas the oxidation of methionine and the deamidation of asparagine and glutamine were set as variable modifications. The false discovery rate was set to less than 0.05, and a minimum length of six amino acids (one peptide per protein) was required for each peptide hit.

The normalized LFQ intensities were processed in Perseus 2.0.11 (Tyanova et al. 2016), filtering for proteins with at least two unique peptides, transforming the intensity values, and averaging three technical replicates via median to treat individual animals (*n* = 14/*n* = 14, including 7 males and females in each group) as the unit of analysis. Only proteins detected in 70% in each sample group were included in the quantitative analyses. A two‐way ANOVA (Genotype/Sex) and normal distribution imputation were applied, with significance set at and an absolute fold change > 0.58 on a logarithmic scale (1.5‐fold change). Pathway analysis was performed using Ingenuity Pathway Analysis (QIAGEN) to determine canonical pathways and disease and function annotations associated with the observed differences in protein profiles. The prediction of the direction of change for a given function or pathway was estimated by a z‐score ≥ 2 (for activation) and ≤ −2 (for inhibition) (Pezzini et al. 2017; Tracz et al. 2021). The Venn diagrams were drawn using BioTools (https://www.biotools.fr/misc/venny).

### 4.3 Cranial Window Surgery for Cortical Window Implantation

We surgically implanted an open‐skull cranial window over the right cortical hemisphere as previously described (Fuhrmann et al. 2010, 2007; Holtmaat et al. 2009; Druart et al. 2021). Mice were anesthetized via intraperitoneal (i.p.) injection of ketamine/xylazine (0.13/0.01 mg/g body weight). Additionally, dexamethasone (0.2 mg/kg) and TEMGESIC (0.05 mg/kg; Reckitt Benckiser Healthcare (UK) Ltd., Great Britain) were administered intraperitoneal injection before surgery. Under anesthesia, we removed a circular piece of the skull over the somatosensory cortex (5 mm diameter) using a dental drill (Schick‐Technikmaster C1; Pluradent; Offenbach, Germany). Special care was taken to leave the dura intact. The dura was covered with PBS immediately to prevent it from drying out. After the craniotomy, we glued a circular coverslip (5 mm diameter, thickness #01) to the skull using dental acrylic (Cyano‐Veneer fast; Heinrich Schein Dental Depot, Munich, Germany) to close the open area. A small metal head‐bar with a screw thread was glued next to the coverslip to allow repositioning of the mouse during longitudinal imaging sessions. The imaging experiments started after a three‐week resting period following cranial window surgery to ensure a stable window and to prevent neuroinflammation.

### 4.4 Two‐Photon In Vivo Imaging

Microglia migration imaging was performed using an upright Zeiss (Carl Zeiss Microscopy GmbH, Jena, Germany) Axio Examiner LSM7MP setup equipped with a Coherent Cameleon Ultra2 two‐photon laser (Coherent, Dieburg, Germany). Microglia fine processes extension analysis was carried out using a TriM Scope II (LaVision BioTec GmbH, Bielefeld, Germany) equipped with a Coherent Cameleon Ultra2 two‐photon laser. Green fluorescent protein (GFP) was excited at 920 nm; the fluorescence emission was bandpass filtered (525/50) and detected by an ultra‐sensitive non‐de‐scanned gallium‐arsenide‐phosphide (GaAsP) detector with large aperture detection optics in proximity to the objective. A Zeiss 20× water immersion objective (NA1.0) or a Nikon 16× water immersion objective (NA0.8) was used for magnification. The standard microscopy table was replaced by a custom‐made table to allow repeated positioning of a living mouse under the microscope. At each imaging time‐point, mice were briefly anesthetized with 0.8% Isoflurane gas anesthesia that lasted for 15–20 min. Image acquisition was performed with the Zeiss ZEN2010 software or Imspector, respectively. To follow the migration or fine processes extension of individual microglia over time, we acquired z‐stacks, 300 μm in size, starting from the cortical surface with a 3 μm *z*‐step size. Image frames were 600 × 600μm in size with 1536 × 1536 pixels in x, y‐direction (migration) or 1243 × 1243 pixels (fine processes extension). At the first imaging time‐point, the distribution of microglia was determined by acquiring a z‐stack as described. Subsequently, a laser lesion was induced by focusing the laser beam in the middle position in x, y‐ and z‐dimension (x: 300 μm, y: 300 μm, z: 150 μm). The wavelength was changed to 800 nm and an output power of 180 ± 5 mW. An area of 10 × 10 μm was scanned for 1.5 s with a pixel dwell time of 1.27 μsec and a pixel size of 0.01 μm/pixel. Subsequently, z‐stacks were acquired every 5 min for fine processes extension analysis and at 4–6 h over a period of 72 h for cell body migration analysis. Care was taken to ensure similar fluorescence levels in space and time. Different cohorts of mice were used for imaging of fine processing extension and the long‐term lesion response. Repositioning over time was achieved by orienting to the vascular pattern under reflected light illumination using a GFP filter set and a metal halide lamp HXP100 (Carl Zeiss Microscopy GmbH, Jena, Germany). Additionally, orienting at the center of the lesion in x‐, y‐ and z‐dimensions was carried out using two‐photon illumination.

### 4.5 Retrieval of Proliferating Microglia in Fixed Tissue After In Vivo Imaging

To retrieve previously imaged migrating and proliferating microglia after in vivo imaging in fixed tissue, mice were injected with EdU (5‐ethynyl‐2′‐deoxyuridine; 25 μg) starting at the first imaging time‐point every 12 h until the end of imaging after 72 h. Immediately after the 72 h imaging time‐point, mice were transcardially perfused with phosphate‐buffered saline (PBS) followed by 4% paraformaldehyde (PFA). The brain was left inside the intact skull, and only the circular coverslip was removed to give access to PFA to the brain for post‐fixation overnight at 4°C. The next day, the mouse head was fixed via the metal head‐bar (see cranial window surgery) in the same plane as imaging had been carried out. The skull over the right cortical hemisphere was carefully removed by using a dental drill. Subsequently, the mouse head was positioned under a vibratome blade (Leica VT1000S, Wetzlar, Germany) to cut the brain into 100 μm‐thick sections. The sections were oriented in the same plane as the imaging had been carried out to generate sections perpendicular to the z‐focusing axis of the microscope. Four to five sections were cut and successively collected in individual wells of a 24‐well plate. Care was taken to keep the topside of the slices up in the PBS‐filled well.

Immunohistochemistry was carried out with a slightly adapted protocol from Gogolla and colleagues (Gogolla et al. 2006): The sections were permeabilized with 2% Triton X‐100 for 12 h at 4°C on a shaker. Slices were washed with 3% BSA in PBS three times for 5 min per wash. The Click‐IT reaction cocktail was prepared as described in the manufacturer's manual (Click‐IT EdU Alexa‐Fluor 647 Imaging Kit, Invitrogen), and 500 μL of the solution was added to each well. Slices were incubated for a period of 60 min, protected from light. Subsequently, the reaction solution was removed, and the slices were washed with 3% BSA in PBS. This procedure avoided an additional staining of microglial *Gfp*, since the very mild staining conditions keep *Gfp* intact. After the staining, slices were mounted on glass objective slides using Fluoromount as mounting medium and glass coverslips. Brain slices were analyzed under epifluorescence using a GFP filter set. Slices that contained lesions were identified, and confocal laser scan images were acquired from the lesion on an inverted Zeiss LSM700 microscope equipped with a 488 nm Ar‐ion and 635 nm HeNe laser to excite GFP and Alexa647. Fluorescence was detected at 500–550 nm (GFP) and at > 650 nm (Alexa 647). A Zeiss 20× objective with NA0.8 or 40× immersion oil objective NA1.3 was used to acquire z‐stacks of microglia around the lesion (Carl Zeiss Microscopy GmbH, Jena, Germany). Images with a frame size of 2048 × 2048 pixels, a pixel size of 0.2 μm/pixel, and a z‐step of 1 μm were acquired in two channels (GFP and Alexa‐Fluor 647). Re‐localization of proliferating microglia was carried out by manually scrolling through the 72‐h in vivo imaging stack and the confocal image stack covering the whole lesion in x, y, and z‐dimension in parallel.

### 4.6 Immunohistochemistry

Mice were deeply anesthetized with an overdose of ketamine‐xylazine and transcardially perfused with PBS followed by 4% paraformaldehyde in PBS. Brains were quickly removed, and the tissue was fixed in 4% PFA overnight. Brains were cut into 100 μm thick coronal slices, and the slices containing the hippocampus were further used for histology. Histology was performed on free‐floating slices. Slices were incubated in Permeabilization Solution (4.3% Normal Goat Serum, 0.43% Triton‐X100, 2.9% Bovine Serum Albumin in PBS) for 2 h at room temperature on a shaker and subsequently incubated with the primary antibodies in Blocking Solution (4.3% Normal Goat Serum, 0.22% Triton‐X100, 2.9% Bovine Serum Albumin in PBS) overnight at room temperature. The following day, the slices were washed with PBS (3×) for 5 min and incubated with the secondary antibodies in Blocking Solution at room temperature for 2 h. DAPI (Sigma‐Aldrich, D9542) was added at a concentration of 1:10000 in the last 20 min of the incubation with the secondary antibodies. After incubation, slices were washed thoroughly with PBS (3×) and finally mounted using Dako mounting medium (Agilent, S3023).

*Primary* antibodies were as follows: rabbit anti‐P2Y12 receptor (1:1000, Synaptic Systems, 476,008), guinea pig anti‐Iba1 (1:500, Synaptic Systems, 234,308).

*Secondary antibodies* used were: goat anti‐guinea pig Alexa Fluor 488 (1:400, Abcam, ab150185), goat anti‐rabbit Alexa Fluor 594 (1:400, Abcam, A11012).

3‐dimensional images were acquired using a Zeiss LSM900 Confocal Microscope equipped with an Airyscan Detector (Carl Zeiss, Oberkochen, Germany). A 63× oil immersion objective was used for all recordings. Images were obtained with a frame size of 225.4 × 225.4 μm with a pixel size of 0.22 μm and a stack comprising 20 μm at a step size of 0.5 μm.

### 4.7 Image Processing and Data Analysis

For fine processes extension analysis, images were processed with the open‐source software Fiji (Schindelin et al. 2012). Ome.tiff files from ImSpector (LaVision Biotec, Bielefeld, Germany) were median filtered and maximum intensity projected. Images were aligned in space and time using the “StackReg” plugin in Fiji (Thevenaz et al. 1998). Individual fine processes were tracked using the Fiji plugin “Manual Tracking” by Fabrice Cordelières, Institut Curie, Orsay (France). The lesion diameter, the Euclidean distance from start to end position, and the total distance that the fine processes traveled were measured by 45 min post lesion. The fine processes' velocity was calculated as the distance traveled divided by time. The meandering index (MI) was calculated by dividing the Euclidean distance by the distance traveled 45 min post lesion.

Microglia cell body migration was analyzed using ZEN, Fiji, and Volocity as analysis software. After acquisition with ZEN, images were median filtered. Subsequently, images were aligned in z,t‐dimension by manually aligning the top and bottom images for each time‐point. Twenty slices, spanning 60 μm and containing the lesion, were maximum intensity projected using the depth color‐coding function in ZEN. Microglia at the bottom were colored in blue, and microglia at the top of the projection were colored in red (Figure S3). These images were exported to Fiji and aligned in *x* and *y* dimensions over the whole imaging time using the “StackReg” plugin. The resulting time series were exported to Volocity (Volocity Ver. 5.5, Perkin Elmer, Rodgau, Germany), which was used for tracking individual microglia. Microglia identified as dividing during tracking were retrieved in fixed brain sections and tested for EdU‐positivity (see “Retrieval of proliferating microglia in fixed tissue after in vivo imaging”). Since imaging was carried out every 4–6 h, all time‐dependent data were binned in eight‐hour bins. The fraction of moving cells was calculated by dividing the number of moving cells at a given time‐point by the total number of cells in the field of view of the 60 μm maximum‐intensity projection. To calculate the distance traveled of microglia toward the lesion, the average distance traveled of all moving cells (translocation > 4 μm) at a given time point was calculated. The velocity was calculated by dividing the distance traveled [μm] of all migrating microglia (translocation > 4 μm) by 72 h. The fraction of proliferating microglia was calculated by dividing the number of dividing cells by the total number of cells in the field of view of the 60 μm maximum‐intensity projections. The distance to the lesion when microglia divided was measured as the Euclidean distance to the center point of the lesion. The MI of migrating microglia was calculated as the Euclidean distance divided by the distance traveled with the lesion center as a reference point.

For reconstruction of Iba1 and P2Y12 receptor, the Imaris Software v2.10.0 (Oxford Instruments plc, Abingdon, United Kingdom) was used. Using the Surfaces function, Iba1 and P2Y12 receptor volumes were reconstructed.

Analysis of the leading process of the microglia was performed using the software Fiji. Stacks obtained 11–12 h after lesion induction were selected, and 10 slices around the lesion center were cut out. These were filtered using the Gaussian Blur (Sigma Radius: 2.00), and subsequently, a maximum intensity projection was prepared. The length of the extended leading process was measured using the line tool, drawing a straight line from the edge of the soma to the end of the leading process (Figure S4b). The analysis was blindly performed by the experimenter.

Microglia motility analysis was performed as previously described (Fuhrmann et al. 2010; Nebeling et al. 2023). Image stacks were registered using the StackReg‐Plugin in ImageJ (Thevenaz et al. 1998) and a maximum intensity projection was generated. The projection of all cells within this stack and their branches was merged with the projections from all recorded timepoints (15 timepoints per recording, delta *t* = 5 min) and registered with the Stack‐Reg Plugin again. Subsequently, the time points were pseudo‐colored in red and green, and the turnover rate of microglial processes was calculated for each time point (Figure S6).

Data analysis was performed blindly by an experimenter without prior knowledge of the experimental conditions. Statistical significance was assumed at *p* < 0.05. Statistical differences in measurements over time were determined using two‐way ANOVA in the software GraphPad Prism, v10.2.2 (GraphPad Software Inc., La Jolla, USA). Statistical differences between multiple experimental groups were carried out by Bonferroni post‐tests. For two‐group comparisons, Student's *t*‐test was used. The key reagents, resources and software can be found in the attached table (Table 1).

**TABLE 1:** Resources table.

Reagent or resource	Source	Identifier
Chemicals, peptides, and recombinant proteins		
5‐ethynyl‐2′‐deoxyuridine	Thermo Fisher Scientific, Waltham, MA, USA	
Ketamine 10%	Serumwerk Bernburg AG, Bernburg, Germany	
Xylazine 2% (Rompun)	Elanco, Monheim am Rhein, Germany	
Temgesic	Reckitt Benckiser Healthcare (UK) Ltd., Great Britain	
Dental acrylic (Cyano‐Veneer fast)	Heinrich Schein Dental Depot, Munich, Germany	
DAPI	Sigma‐Aldrich, St. Louis, USA, MS	D9542
Rabbit anti‐P2Y12 receptor antibody	Synaptic Systems GmBH, Göttingen, Germany	476,008
Guinea pig anti‐Iba1 antibody	Synaptic Systems GmBH, Göttingen, Germany	234,308
Goat anti‐guinea pig Alexa Fluor 488 antibody	Abcam Ltd., Cambridge, United Kingdom	ab150185
Goat anti‐rabbit Alexa Fluor 594 antibody	Abcam Ltd., Cambridge, United Kingdom	A11012
Dako mounting medium	Agilent, Santa Clara, USA, CA	S3023
Critical commercial assays		
Click‐iT EdU Imaging Kit	Thermo Fisher Scientific, Waltham, MA, USA	
Deposited data		
Proteomics data	MassIVE: MSV000095652	
Experimental models: Organisms/strains		
*Cx3cr1* ^*gfp*^ mice	Provided by Steffen Jung (Weizmann Institute of Science, Rehovot, Israel) Jung et al. (2000)	RRID:IMSR_JAX:031582
*Cx3cr1* ^*creERT2*^ mice	Provided by Steffen Jung (Weizmann Institute of Science, Rehovot, Israel) Jung et al. (2000)	RRID:IMSR_JAX:020940
*Cx3cr1* ^*gfp*^ *Ccr2* ^*−/−*^ mice	Generated by Daniel R. Engel and Christian Kurts, *Ccr2* ^*−/−*^ mice^38^	
Oligonucleotides		
Forward: CAG TGA TGC TCT TGG GCT TCC	Kindly provided by Rohini Kuner and Dunja Baumgartl‐Ahlert, University of Heidelberg	
Reverse 1: TCA GTG TTT TCT CCC GCT TGC	Kindly provided by Rohini Kuner and Dunja Baumgartl‐Ahlert, University of Heidelberg	
Reverse 2: ATC AAC GTT TTG TTT TCG GA	Kindly provided by Rohini Kuner and Dunja Baumgartl‐Ahlert, University of Heidelberg	
IMR3945 5′‐TTCACG TTCGGTCTGGTGGG‐3′	Provided by Steffen Jung (Weizmann Institute of Science, Rehovot, Israel)	
IMR3946 5′‐GGTTCCTAGTGGAGCTAGGG‐3′	Provided by Steffen Jung (Weizmann Institute of Science, Rehovot, Israel)	
IMR3947 5′‐GAT CACTCTCGGCATGGACG‐3′	Provided by Steffen Jung (Weizmann Institute of Science, Rehovot, Israel) Jung et al. (2000)	
P1: 5′‐TAA ACC TGG TCA CCA CAT GC‐3′	Provided by Daniel R. Engel and Christian Kurts	
P2: 5′‐TCA CTT ACT TTA CAA CCC AAC C‐3′	Provided by Daniel R. Engel and Christian Kurts	
P3: 5′‐GGA GTA GAG TGG AGG CAG GA‐3′	Provided by Daniel R. Engel and Christian Kurts	
P4: 5′‐GAA GGC GAT AGA AGG CGA T‐3′	Provided by Daniel R. Engel and Christian Kurts	
Software and algorithms		
Proteome Discoverer, version 2.5	Thermo Fisher Scientific, Waltham, MA, USA	
Perseus, version 2.0.11	Tyanova et al. (2016)	
Ingenuity Pathway Analysis	Quiagen GmBH, Hilden, Germany	
ZEN2010	Carl Zeiss Microscopy GmbH, Jena, Germany	
Imaris v2.10.0	Oxford Instruments plc, Abingdon, United Kingdom	
ImSpector	LaVision Biotec, Bielefeld, Germany	
Fiji	Schindelin et al. (2012)	
Volocity version 5.5	Perkin Elmer, Rodgau, Germany	
GraphPad Prism, version 10.2.2	GraphPad Software Inc., La Jolla, USA	
Other		
nano‐LC–Thermo Q Exactive Plus Orbi‐Trap mass spectrometer	Thermo Fisher Scientific, Waltham, MA, USA	
Axio examiner LSM7MP Setup	Carl Zeiss Microscopy GmbH, Jena, Germany	
Coherent Cameleon Ultra2 two‐photon laser	Coherent, Dieburg, Germany	
TriM Scope II	LaVision BioTec GmbH, Bielefeld, Germany	
16× water immersion objective (NA0.8)	Nikon, Minato, Japan	
20× water immersion objective (NA1.0)	Carl Zeiss Microscopy GmbH, Jena, Germany	
Metal halide lamp HXP100	Carl Zeiss Microscopy GmbH, Jena, Germany	
Ultra‐sensitive non‐de‐scanned gallium‐arsenide‐phosphide (GaAsP) detector	Carl Zeiss Microscopy GmbH, Jena, Germany	
Leica VT1000S Vibratome	Leica Biosystems, Wetzlar, Germany	
Inverted LSM700 microscope	Carl Zeiss Microscopy GmbH, Jena, Germany	
Dental drill (Schick‐Technikmaster C1)	Pluradent; Offenbach, Germany	

## Author Contributions

J.W., C.H., H.A., and M.F. prepared figures and wrote the manuscript with input from all authors. J.W., C.H., M.F., L.S., and K.K. carried out surgeries and recorded in vivo imaging data. S.C. and J.S. performed colony management and generated tissue samples. J.S., J.W., and C.H. performed histology and microscopic analysis. R.S., K.L., and M.Ł. acquired proteomics data. J.W., C.H., H.A., and M.Ł. analyzed data. C.K., D.R.E., and J.H. provided lab space, reagents, and discussed the manuscript. M.Ł. and M.F. conceived, planned, and supervised the project.

## Funding

This work was supported by European Research Council, MicroSynCom 865618, ERA‐NET Neuron, MicroSchiz, Deutsche Forschungsgemeinschaft, SFB1089 C01, SFB1089 B06, SPP2395, FOR5427 (466687329) SP4; EN984/16‐1, 18‐1 and 19‐1, TR332 (449437943) A3, INST 20876/486‐1, Ministry of Culture and Science of the State of North Rhine‐Westphalia, iBehave network, Academy of Finland, #318857, ERA‐NET Co‐Fund project, JTC2017, MicroSynDep Consortium, Boehringer Ingelheim Fonds PhD Fellowship.

## Ethics Statement

All experiments involving animals were conducted in accordance with the internal regulation of the DZNE and the government of North‐Rhine‐Westphalia (License: Az 84‐02.04.2017.A098).

## Conflicts of Interest

The authors declare no conflicts of interest.

## Supporting information


**Data S1:** Supplement captions.

**Video S1:** Microglia migration. Time‐lapse video of microglia migration after a laser lesion over a period of 72 h. In the left panel, microglia are depicted in green. The right panel shows individual microglia cells as white dots moving toward the lesion over the entire imaging period. Interestingly, previously sessile microglia migrate toward the lesion. Scale bar: 50 μm.

**Video S2:** Microglia filopodia extension. Microglia fine‐process extension upon induction of a laser lesion within the first 75 min was acquired in five‐minute time intervals. The left panel shows an overview image of microglia in proximity and further away from the lesion spot. The right panel shows a zoom‐in of the time‐lapse of individual microglia fine processes extension. The blue line exemplarily illustrates a tracked microglial fine process. Scale bars: 35 μm (left), 10 μm (right).

**Video S3:** Microglia division. Exemplary in vivo time‐lapse of a dividing microglial cell after a laser lesion over a time period of 72 h. The brain slices were fixed in PFA after 72 h. In vivo imaged microglia (top right) were re‐localized in the fixed brain slices, and immunohistochemical staining was performed for the proliferation marker EdU (red, bottom left). Additionally, GFP in microglia is displayed (green, top right), and the images are depicted individually and merged (bottom right) in order to show EdU‐positive proliferating cells. Red and green arrows indicate dividing microglia; the blue arrow indicates a non‐dividing microglial cell. Scale bar: 25 μm.

**Table S1:** All identified and differentially regulated proteins in the cortex of *Cx3cr1* ^*−/−*^ and *Cx3cr1* ^*−/+*^ mice. All 2668 identified proteins are depicted, including their accession numbers, description of the protein function, False Discovery Rate (FDR), *q*‐values, Posterior Error Probability (PEP) Score, Coverage, Peptide number out of which the number of unique peptides for each protein was identified, and information on the protein characteristics. The information on gender and genotype is provided: F_HM—female homozygous, F_HT—female heterozygous, M_HM—male homozygous, M_HT‐ male heterozygous.

**Table S2:** Differentially abundant proteins among *Cx3cr1* ^*−/−*^ and *Cx3cr1* ^*−/+*^ mice. From all detected proteins, 152 proteins that differ in their abundance between conditions were identified utilizing a two‐way ANOVA using Genotype and Sex as independent factors. Only proteins with at least two unique peptides and an FDR of < 0.01 for identification were included. Significance was defined by an ANOVA *p*‐value < 0.05 and a minimum absolute fold change of 0.58 on a logarithmic scale (1.5‐fold change).

**Table S3:** Ingenuity canonical pathways analysis. Based on the previously identified differentially abundant proteins, Ingenuity Pathway Analysis (IPA) was conducted to predict pathways that are altered in the cortex of the *Cx3cr1* ^*−/−*^ compared to the *Cx3cr1* ^*−/+*^ mice. A prediction of pathway activity is given (based on *z*‐score ∣ ≥ 2∣).

**Table S4:** Comparison of identified DEPs with the microglial proteome. Comparison of differentially abundant proteins (DEPs) identified in this study with the microglial proteome dataset from Lloyd et al. (2024). DEP_ID_LFQ: Differentially abundant proteins identified in the cortex in this study. Shared ID_LFQ_Lloyd et al. (2024): Overlapping protein identifiers between the current study and Lloyd et al. (2024). Unique ID_LFQ cortex: Protein identifiers unique to the cortical dataset in this study.

**Figure S1:** Comparative and functional categories analysis of *Cx3cr1* ^*+/−*^ and *Cx3cr1* ^*−/−*^ proteomes in the cortex and their comparison with published microglial transcriptomic datasets.
(a) The PCA was updated using the high‐confidence subset of 152 DEPs. This supervised approach revealed that genotype is the dominant driver of variance (PC1 = 55.7%), while PC2 (14.7%) effectively captures the sex‐specific divergence. (b) Venn diagram analysis between 152 Differentially Abundant Proteins (DEPs) and the differentially expressed genes, DEGs reported in (Gyoneva et al. 2019; Zhang et al. 2014; Butovsky et al. 2014; Hickman et al. 2013). (c) Canonical pathway analysis of DEPs with depiction of identified supra‐categories. Only categories with a prediction of pathway activity based on a z‐score of ∣ ≥ 2∣ are shown. (d) Hierarchical clustering (Spearman correlation, average linkage) of the significant protein signature demonstrated high homogeneity, with 92.6% (25/27) of biological replicates clustering correctly by genotype.

**Figure S2:** CX3CR1‐independent parameters. (a) Average lesion diameter of *Cx3cr1* ^*gfp/+*^ and *Cx3cr1* ^*gfp/gfp*^ mice is unchanged (*gfp/+*: 108.3 ± 12.1 μm; *gfp/gfp*: 125.0 ± 5.2 μm; *n* = 5–6 mice/group, *p* > 0.05). (b) Unaltered average recruitment radius of migrating microglia comparing *Cx3cr1* ^*gfp/+*^ and *Cx3cr1* ^*gfp/gfp*^ mice (*Cx3cr1* ^*gfp/+*^: 150.8 ± 9.8 μm; *Cx3cr1* ^*gfp/gfp*^: 156.2 ± 7.1 μm; *n* = 5–6 mice/group, *p* > 0.05). (c) The meandering index of microglia migrating to the lesion reveals a high degree of directionality and was unchanged between *Cx3cr1* ^*gfp/+*^ and *Cx3cr1* ^*gfp/gfp*^ mice (*Cx3cr1* ^*gfp/+*^: 0.935 ± 0.018; *Cx3cr1* ^*gfp/gfp*^: 0.929 ± 0.012; *n* = 5–6 mice/group, *p* > 0.05). (d) Unchanged maximum recruitment radius between *Cx3cr1* ^*gfp/+*^ and *Cx3cr1* ^*gfp/gfp*^ mice (*Cx3cr1* ^*gfp/+*^: 248 ± 15 μm; *Cx3cr1* ^*gfp/gfp*^: 259 ± 13 μm; *n* = 5–6 mice/group, *p* > 0.05). (e) Microglia density before the laser lesion was unchanged between *Cx3cr1* ^*gfp/+*^ and *Cx3cr1* ^*gfp/gfp*^ mice (*Cx3cr1* ^*gfp/+*^: 7801 ± 567 μm; *Cx3cr1* ^*gfp/gfp*^: 7593 ± 838 μm; *n* = 5–6 mice/group, *p* > 0.05). (f) Fraction of migrating cells in the analyzed volume is not significantly different between the two genotypes (*Cx3cr1* ^*gfp/+*^: 29.7% ± 5.7%; *Cx3cr1* ^*gfp/gfp*^: 22.2% ± 4.6%; *n* = 5–6 mice/group, *p* > 0.05).

**Figure S3:** Color‐coded depth projection. (a) Maximum intensity projection of a 60 μm depth‐spanning z‐stack containing GFP‐labeled microglia acquired with a z‐step of 3 μm distance. Upper panel x, y‐projection, lower panel x, z‐projection. (b) Color‐coded maximum intensity projections (x, y and x, z) of the same z‐stack. Different depths of individual microglia cells are clearly visualized.

**Figure S4:** Microglia morphology change after migration initiation. (a) Maximum Intensity projection of a lesion (30 μm) indicating the initiation of microglia migration toward a lesion with a temporal resolution of 3 h after lesion initiation up to 12 h post lesion induction. Scale bars = 50 μm (b) Zoom‐In focusing tracking the morphological changes of one microglial cell over the first 12 h after lesion induction. The formation of one leading process is illustrated over time (arrowheads). Scale bars = 20 μm. (c) Zoom‐In focusing on non‐migrating microglial cells 12 h after lesion induction (arrowheads). Scale bar = 20 μm. (d) The length of the leading process (magenta line indicated in b) does not differ between *Cx3cr1* ^*gfp/+*^ (47 ± 3 μm) compared to *Cx3cr1* ^*gfp/gfp*^ (46 ± 2 μm) mice 12 h after lesion induction (determined using an unpaired *t*‐test). *N* = 5 *Cx3cr1* ^*gfp/+*^ and 4 *Cx3cr1* ^*gfp/gfp*^ mice. A total of *n* = 142 migrating microglia in *Cx3cr1* ^*gfp/+*^ mice, compared to 105 migrating microglia in *Cx3cr1* ^*gfp/gfp*^ mice, was measured. Data are displayed as mean ± SEM.

**Figure S5:** P2Y12 receptor expression. (a) Histology was conducted in the cortex to compare the expression of microglial markers Iba1 and P2Y12R in the cortex of *Cx3cr1* ^*gfp/+*^ and *Cx3cr1* ^*gfp/*gfp^ mice. (b) Exemplary images showing the microglial markers Iba1 and P2Y12R, as well as DAPI for cell nuclei. Zoom images show an individual cell and the volume reconstructions of both microglial markers in IMARIS software. Scale Bars = 20 μm (Over‐Views), 10 μm (Zoom‐Ins). (c) Assessing Iba1 (top) and P2Y12R (bottom) volume and the (d) P2Y12R/Iba1 volume ratio (Iba1: *Cx3cr1* ^*gfp/+*^: 6.7% ± 0.38%, *Cx3cr1* ^*gfp/gfp*^: 5.1% ± 0.33%; P2Y12: *Cx3cr1* ^*gfp/+*^: 7.4% ± 0.46%, *Cx3cr1* ^*gfp/gfp*^: 6.4% ± 0.43%; P2Y12/Iba1: *Cx3cr1* ^*gfp/+*^: 1.1 ± 0.10, *Cx3cr1* ^*gfp/gfp*^: 1.3 ± 0.04). Data in (c and d) are depicted as mean ± SEM. Comparisons were conducted using an unpaired *t*‐test. Significance was assumed if *p* < 0.05. *N* = 5 *Cx3cr1* ^*gfp*/+^ (1 male, 4 females) *N* = 5 *Cx3cr1* ^*gfp*/*gfp*^ (3 males, 2 females) mice, *n* = 4 FOVs/mouse.

**Figure S6:** Microglia fine processes turnover rate. (a) Exemplary images of microglia fine processes turnover in the cortex comparing *Cx3cr1* ^*gfp/+*^ and *Cx3cr1* ^*gfp/gfp*^ mice. (b) Microglia fine processes turnover rate (TOR) was calculated as the number (absolute pixel value) of lost, N_lost_ (red), and newly gained, N_gained_ (green) pixels divided by the sum of all pixels within a region of interest. (c) Microglia fine processes gain, loss, and turnover rate of *Cx3cr1* ^*gfp/+*^ and *Cx3cr1* ^*gfp/gfp*^ mice (*n* = 4 stacks per mouse, *N* = 4 mice/group, 3 males, one female per group), unpaired *t*‐test, (two‐tailed). Error bars: SEM.

**Figure S7:** Overview of all mouse models and the genotyping. (a) Illustration of the genomic *Cx3cr1* locus in which Cx3cr1 is partially replaced by a *CreERT2* cassette or a *gfp*. (b) Illustration of the genomic *Ccr2* locus and the *Ccr2* knockout allele with various restriction sites EcoR1 (E), BamH1 (Bm), Xba1 (X), and Primer location P1‐P4. Primer P1 lies in the non‐coding region before the ATG start‐codon. P2 is located at the exon‐intron boundary. This part was not included in the targeting construct. P3 is located at the BamH1 restriction site, where the neomycin resistance was introduced. P4 is part of the neomycin resistance. (c) Exemplary images of agarose gels for the different genotypes of *Ccr2::Cx3cr1* ^*CreERT2*^ mice. *Cx3cr1* ^*−/−*^ yield one ca. 700 bp long fragment, *Cx3cr* ^*+/+*^yield one ~400 bp fragment, and *Cx3cr1* ^*+/−*^ yield two fragments of both sizes. (d) Exemplary images of agarose gels for the different genotypes of *Ccr2::Cx3cr1* ^*gfp*^ mice. To determine the zygosity for *Cx3cr1* ^*gfp*^, the PCR resulted in a 970 bp fragment for the wild‐type allele and a 1.2 kb fragment for the knockout allele. Heterozygous mice display both bands. To determine *Ccr2* zygosity, primer P1‐4 was used. P1 and P3 amplify a 400 bp exclusively from the wild‐type allele, while P1 and P4 yield a 1.2 kb fragment exclusively from the *Ccr2* knockout allele. Primer P1/P2 amplifies a 1.4 kb and a 3.2 kb fragment from the wild‐type and knockout allele, respectively. Moreover, P2 is located at the exon‐intron boundary of the *Ccr2* gene that was not part of the targeting construct.

**Figure S8:** Enlarged version of Figure 1e for improved readability.

## Data Availability

The data that support the findings of this study are openly available in Mass Spectrometry Interactive Virtual Environment (MassIVE) at https://massive.ucsd.edu/ProteoSAFe/private‐dataset.jsp?task=06782d6f1b0a4a14bc654fc1811a10d2, reference number MSV000095652.
