# Supplementary material for: CX3CR1 Modulates Migration of Resident Microglia Towards Cortical Laser‐Induced Lesions
Source: Glia. 2026 Aug 15;74(10):e70207. doi: 10.1002/glia.70207 (PMC13476746; doi:10.1002/glia.70207)
Supplement: Supplementary file 1 [file GLIA-74-0-s009.docx]

**CX3CR1 modulates the migration of resident microglia towards cortical laser-induced lesions**

Jens Wagner^1,#^, Henrike Antony^1,#^, Cornelia Hoyer^1^, Kristiina Lundgrén^5^, Rabah Soliymani^5^, Sophie Crux^1^, Lena Justus^1^, Kevin Keppler^1,3^, Julia Steffen^1^, Christian Kurts^4^, Daniel R. Engel^7^, Jochen Herms^2,3^, Maciej Łałowski^5,6,^* and Martin Fuhrmann^1,^*

^1^German Center for Neurodegenerative Diseases (DZNE), Bonn, Germany,

^2^German Center for Neurodegenerative Diseases (DZNE), Munich, Germany,

^3^Center of Neuropathology and Prion Research, Ludwig Maximilians University, Munich, Germany,

^4^Institute of Molecular Medicine and Experimental Immunology, University of Bonn, Germany,

^5^Helsinki Institute of Life Science (HiLIFE), Meilahti Proteomics Unit (MPU), University of Helsinki, Helsinki, Finland,

^6^Department of Gene Expression, Institute of Molecular Biology and Biotechnology, Faculty of Biology, Adam Mickiewicz University, Poznań, Poland,

^7^Institute of Experimental Immunology and Imaging, University Duisburg-Essen, Germany

^#^equal contribution

*equal contribution

Correspondence: [martin.fuhrmann@dzne.de](mailto:martin.fuhrmann@dzne.de)

**Supplementary Material**

**Supplementary Figures**

**Supplementary Figure 1. Comparative and functional categories analysis of *Cx3cr1^+/–^* and *Cx3cr1^–/–^* proteomes in the cortex and their comparison with published microglial transcriptomic datasets.**

**(a)** The PCA was updated using the high-confidence subset of 152 DEPs. This supervised approach revealed that genotype is the dominant driver of variance (PC1 = 55.7%), while PC2 (14.7%) effectively captures the sex-specific divergence. **(b)** Venn diagram analysis between 152 Differentially Abundant Proteins (DEPs) and the differentially expressed genes, DEGs reported in [22-25]. **(c)** Canonical pathway analysis of DEPs with depiction of identified supra-categories. Only categories with a prediction of pathway activity based on a z-score of ⏐≥2⏐ are shown. **(d)** Hierarchical clustering (Spearman correlation, average linkage) of the significant protein signature demonstrated high homogeneity, with 92.6% (25/27) of biological replicates clustering correctly by genotype.

**Supplementary Figure 2. CX3CR1-independent parameters.**

**(a)** Average lesion diameter of *Cx3cr1^gfp/+^* and *Cx3cr1^gfp/gfp^* mice is unchanged (*gfp/+*: 108.3±12.1µm; *gfp/gfp*: 125.0±5.2µm; n=5-6 mice/group, p>0.05). **(b)** Unaltered average recruitment radius of migrating microglia comparing *Cx3cr1^gfp/+^* and *Cx3cr1^gfp/gfp^* mice (*Cx3cr1^gfp/+^*: 150.8±9.8µm; *Cx3cr1^gfp/gfp^*: 156.2±7.1µm; n=5-6 mice/group, p>0.05). **(c)** The meandering index of microglia migrating to the lesion reveals a high degree of directionality and was unchanged between *Cx3cr1^gfp/+^* and *Cx3cr1^gfp/gfp^* mice (*Cx3cr1^gfp/+^*: 0.935±0.018; *Cx3cr1^gfp/gfp^*: 0.929±0.012; n=5-6 mice/group, p>0.05). **(d)** Unchanged maximum recruitment radius between *Cx3cr1^gfp/+^* and *Cx3cr1^gfp/gfp^* mice (*Cx3cr1^gfp/+^*: 248±15µm; *Cx3cr1^gfp/gfp^*: 259±13µm; n=5-6 mice/group, p>0.05). **(e)** Microglia density before the laser lesion was unchanged between *Cx3cr1^gfp/+^* and *Cx3cr1^gfp/gfp^* mice (*Cx3cr1^gfp/+^*: 7801±567µm; *Cx3cr1^gfp/gfp^*: 7593±838µm; n=5-6 mice/group, p>0.05). **(f)** Fraction of migrating cells in the analyzed volume is not significantly different between the two genotypes (*Cx3cr1^gfp/+^*: 29.7±5.7%; *Cx3cr1^gfp/gfp^*: 22.2±4.6%; n=5-6 mice/group, p>0.05).

**Supplementary Figure 3. Color-coded depth projection.**

**(a)** Maximum intensity projection of a 60µm depth-spanning z-stack containing GFP-labeled microglia acquired with a z-step of 3µm distance. Upper panel x, y-projection, lower panel x, z-projection. **(b)** Color-coded maximum intensity projections (x, y and x, z) of the same z-stack. Different depths of individual microglia cells are clearly visualized.

**Supplementary Figure 4. Microglia Morphology Change after Migration Initiation.**

**(a)** Maximum Intensity projection of a lesion (30µm) indicating the initiation of microglia migration towards a lesion with a temporal resolution of 3h after lesion initiation up to 12h post lesion induction. Scale bars = 50µm **(b)** Zoom-In focusing tracking the morphological changes of one microglial cell over the first 12h after lesion induction. The formation of one leading process is illustrated over time (arrowheads). Scale bars = 20µm. **(c)** Zoom-In focusing on non-migrating microglial cells 12h after lesion induction (arrowheads). Scale bar = 20µm. **(d)** The length of the leading process (magenta line indicated in b) does not differ between *Cx3cr1^gfp/+^* (47±3µm) compared to *Cx3cr1^gfp/gfp^* (46±2µm) mice 12h after lesion induction (determined using an unpaired t-test). N=5 *Cx3cr1^gfp/+^* and 4 *Cx3cr1^gfp/gfp^* mice. A total of n=142 migrating microglia in *Cx3cr1^gfp/+^* mice, compared to 105 migrating microglia in *Cx3cr1^gfp/gfp^* mice, was measured. Data are displayed as mean±SEM.

**Supplementary Figure 5. P2Y12 receptor expression.**

(a) Histology was conducted in the cortex to compare the expression of microglial markers Iba1 and P2Y12R in the cortex of *Cx3cr1^gfp/+^* and *Cx3cr1^gfp/^*^gfp^ mice. (b) Exemplary images showing the microglial markers Iba1 and P2Y12R, as well as DAPI for cell nuclei. Zoom images show an individual cell and the volume reconstructions of both microglial markers in IMARIS software. Scale Bars = 20μm (Over-Views), 10μm (Zoom-Ins). **(c)** Assessing Iba1 (top) and P2Y12R (bottom) volume and the **(d)** P2Y12R/Iba1 volume ratio (Iba1: *Cx3cr1^gfp/+^*: 6.7±0.38%, *Cx3cr1^gfp/gfp^*: 5.1±0.33%; P2Y12: *Cx3cr1^gfp/+^*: 7.4±0.46%, *Cx3cr1^gfp/gfp^*: 6.4±0.43%; P2Y12/Iba1: *Cx3cr1^gfp/+^*: 1.1±0.10, *Cx3cr1^gfp/gfp^*: 1.3±0.04). Data in (c and d) are depicted as mean±SEM. Comparisons were conducted using an unpaired t-test. Significance was assumed if p<0.05. N=5 *Cx3cr1^gfp^*^/+^ (1 male, 4 females) N=5 *Cx3cr1^gfp^*^/^*^gfp^* (3 males, 2 females) mice, n=4 FOVs/mouse.

**Supplementary Figure 6. Microglia Fine Processes Turnover Rate.**

**(a)** Exemplary images of microglia fine processes turnover in the cortex comparing *Cx3cr1^gfp/+^* and *Cx3cr1^gfp/gfp^* mice. **(b)** Microglia fine processes turnover rate (TOR) was calculated as the number (absolute pixel value) of lost, N_lost_ (red), and newly gained, N_gained_ (green) pixels divided by the sum of all pixels within a region of interest. **(c)** Microglia fine processes gain, loss, and turnover rate of *Cx3cr1^gfp/+^* and *Cx3cr1^gfp/gfp^* mice (n=4 stacks per mouse, N=4 mice/group, 3 males, one female per group), unpaired t-test, two-tailed). Error bars: SEM.

**Supplementary Figure 7. Overview of all mouse models and the genotyping.**

**(a)** Illustration of the genomic *Cx3cr1* locus in which Cx3cr1 is partially replaced by a *CreERT2* cassette or a *gfp*. **(b)** Illustration of the genomic *Ccr2* locus and the *Ccr2* knockout allele with various restriction sites EcoR1 (E), BamH1 (Bm), Xba1 (X), and Primer location P1-P4. Primer P1 lies in the non-coding region before the ATG start-codon. P2 is located at the exon-intron boundary. This part was not included in the targeting construct. P3 is located at the BamH1 restriction site, where the neomycin resistance was introduced. P4 is part of the neomycin resistance. **(c)** Exemplary images of agarose gels for the different genotypes of *Ccr2::Cx3cr1^CreERT2^* mice. *Cx3cr1^–/–^* yield one ca. 700bp long fragment, *Cx3cr^+/+^*yield one ~400bp fragment, and *Cx3cr1^+/–^* yield two fragments of both sizes. **(d)** Exemplary images of agarose gels for the different genotypes of *Ccr2::Cx3cr1^gfp^* mice. To determine the zygosity for *Cx3cr1^gfp^*, the PCR resulted in a 970 bp fragment for the wild-type allele and a 1.2kb fragment for the knockout allele. Heterozygous mice display both bands. To determine *Ccr2* zygosity, primer P1-4 was used. P1 and P3 amplify a 400bp exclusively from the wild-type allele, while P1 and P4 yield a 1.2kb fragment exclusively from the *Ccr2* knockout allele. Primer P1/P2 amplifies a 1.4kb and a 3.2kb fragment from the wild-type and knockout allele, respectively. Moreover, P2 is located at the exon-intron boundary of the *Ccr2* gene that was not part of the targeting construct.

**Supplementary Figure 8. Enlarged version of Figure 1e for improved readability.**

**Supplementary Videos**

**Supplementary Video 1. Microglia Migration.**

Time-lapse video of microglia migration after a laser lesion over a period of 72h. In the left panel, microglia are depicted in green. The right panel shows individual microglia cells as white dots moving towards the lesion over the entire imaging period. Interestingly, previously sessile microglia migrate towards the lesion. Scale bar: 50µm.

**Supplementary Video 2. Microglia Filopodia Extension.**

Microglia fine-process extension upon induction of a laser lesion within the first 75min was acquired in five-minute time intervals. The left panel shows an overview image of microglia in proximity and further away from the lesion spot. The right panel shows a zoom-in of the time-lapse of individual microglia fine processes extension. The blue line exemplarily illustrates a tracked microglial fine process. Scale bars: 35µm (left), 10µm (right).

**Supplementary Video 3. Microglia Division.**

Exemplary *in vivo* time-lapse of a dividing microglial cell after a laser lesion over a time period of 72h. The brain slices were fixed in PFA after 72h. *In vivo* imaged microglia (top right) were re-localized in the fixed brain slices, and immunohistochemical staining was performed for the proliferation marker EdU (red, bottom left). Additionally, GFP in microglia is displayed (green, top right), and the images are depicted individually and merged (bottom right) in order to show EdU-positive proliferating cells. Red and green arrows indicate dividing microglia; the blue arrow indicates a non-dividing microglial cell. Scale bar: 25µm.

**Supplementary Tables**

**Supplementary Table 1. All identified proteins in the cortex of *Cx3cr1^–/–^* and *Cx3cr1^–/+^* mice.**

All 2668 identified proteins are depicted, including their accession numbers, description of the protein function, False Discovery Rate (FDR), q-values, Posterior Error Probability (PEP) Score, Coverage, Peptide number out of which the number of unique peptides for each protein was identified, and information on the protein characteristics. The information on gender and genotype is provided: F_HM - female homozygous, F_HT - female heterozygous, M_HM - male homozygous, M_HT- male heterozygous.

**Supplementary Table 2. Differentially abundant proteins among *Cx3cr1^–/–^* and *Cx3cr1^–/+^* mice.**

From all detected proteins, 152 proteins that differ in their abundance between conditions were identified utilizing a two-way ANOVA using Genotype and Sex as independent factors. Only proteins with at least two unique peptides and an FDR of <0.01 for identification were included. Significance was defined by an ANOVA p-value < 0.05 and a minimum absolute fold change of 0.58 on a logarithmic scale (1.5-fold change).

**Supplementary Table 3. Ingenuity Canonical Pathways Analysis.**

Based on the previously identified differentially abundant proteins, Ingenuity Pathway Analysis (IPA) was conducted to predict pathways that are altered in the cortex of the *Cx3cr1^–/–^* compared to the *Cx3cr1^–/+^* mice. A prediction of pathway activity is given (based on z-score ⏐≥2⏐.

**Supplementary Table 4. Comparison of identified DEPs with the microglial proteome.**
Comparison of differentially abundant proteins (DEPs) identified in this study with the microglial proteome dataset from Lloyd et al. (2024). DEP_ID_LFQ: Differentially abundant proteins identified in the cortex in this study. Shared ID_LFQ_Lloyd et al. (2024): Overlapping protein identifiers between the current study and Lloyd et al. (2024). Unique ID_LFQ cortex: Protein identifiers unique to the cortical dataset in this study.
